# Pierceable, Water‐Resistant, and Transparent Nanofilm Electrodes Comprising Carbon Nanotubes for Long‐Term Monitoring of Plant Electrophysiology

**DOI:** 10.1002/advs.202522824

**Published:** 2026-03-23

**Authors:** Yusuke Hori, Tatsuhiro Horii, Shinji Masuda, Toshinori Fujie

**Affiliations:** ^1^ Department of Life Science and Technology Institute of Science Tokyo Yokohama Japan; ^2^ Research Center for Autonomous Systems Materialogy (ASMat) Institute of Integrated Research (IIR) Institute of Science Tokyo Yokohama Japan; ^3^ Institute of Biomedical Engineering (BME) Institute of Science Tokyo Yokohama Japan

**Keywords:** conformal electrodes, flexible bioelectronics, plant electrophysiology, polymeric nanofilms, trichomes, water‐resistant electrodes

## Abstract

Trichomes are found on the surfaces of many crops, making it challenging to design sensors that monitor plant conditions without interfering with their natural functions. Notably, conventional and advanced electrodes developed for plant electrophysiology have not overcome this challenge. Herein, we present thin‐film composite electrodes that are transparent, water‐vapor‐permeable, water‐resistant, non‐invasive, pierceable by trichomes, and conformal to the complex surfaces of leaves, ensuring low contact impedance without the need for adhesives. The bilayer electrodes (referred to as “nanofilm electrodes”) are composed of single‐walled carbon nanotubes (SWCNTs) deposited on poly(styrene‐*b*‐butadiene‐*b*‐styrene), and the thickness is varied (70–480 nm) to evaluate its influence. Furthermore, the sensing performance and long‐term stability are compared with those of commercial wet electrodes and conventional poly(3,4‐ethylenedioxythiophene):poly(4‐styrenesulfonate) electrodes. The proposed SWCNT nanofilm electrodes enable stable detection of electrophysiological changes in leaves for over 2 months, as well as light‐induced biopotential signals under chemical stress, highlighting their potential for long‐term, real‐time plant health monitoring in smart agriculture systems.

## Graphical abstract

Ultra‐flexible electrodes composed of single‐walled carbon nanotubes on a polymeric substrate exhibit excellent transparency, water resistance, and conformability to hairy surfaces. These non‐invasive nanofilms are easily pierced by trichomes, overcoming the structural barriers in plant electrophysiology. This design enables stable, long‐term signal detection without interference from rainfall, offering a robust solution for real‐time plant health monitoring.

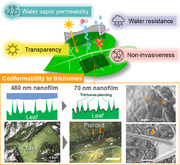

## 1 Introduction

**FIGURE 1:**
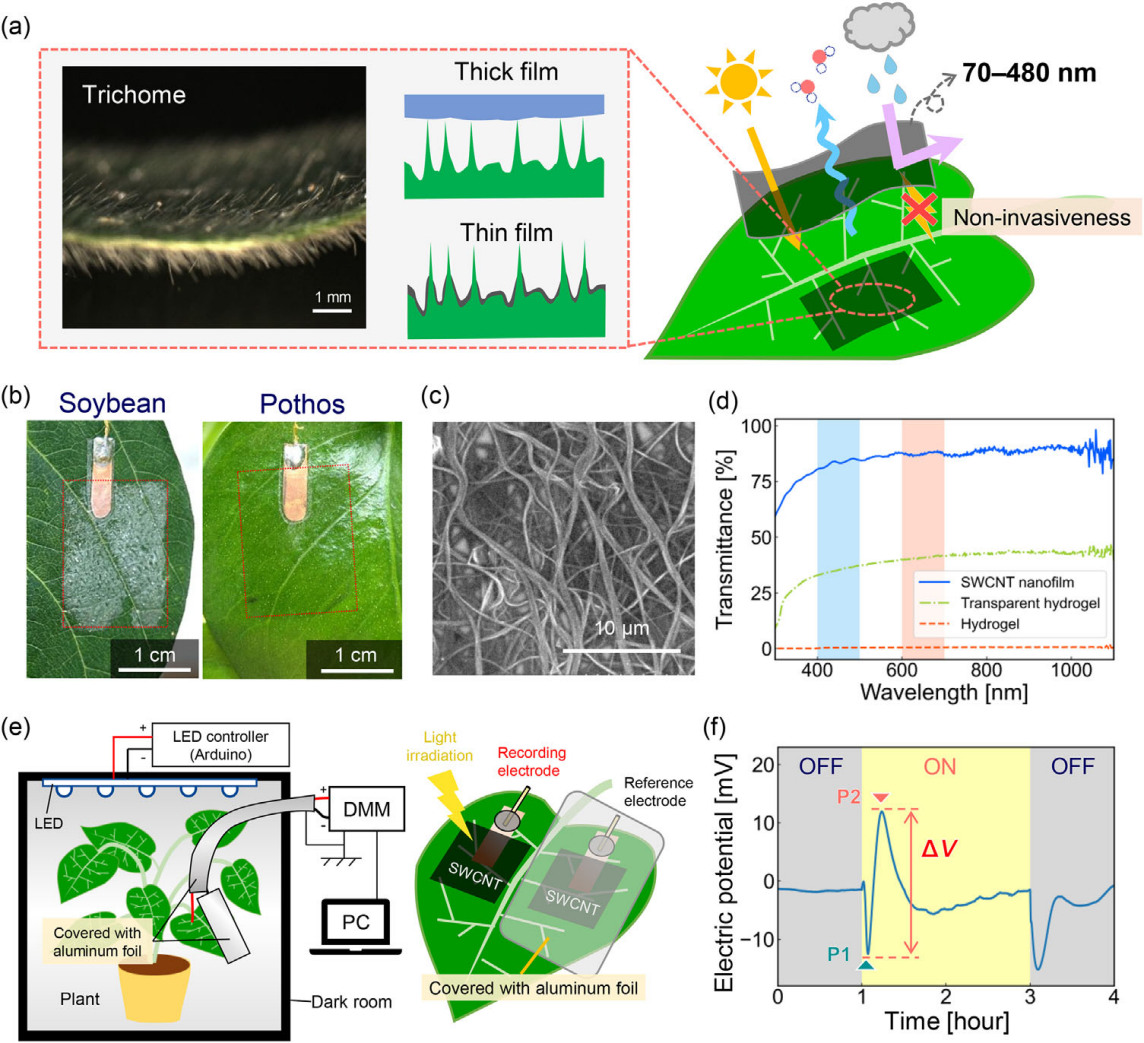
A flexible and transparent plant surface electrode. (a) A schematic illustration demonstrating the application of thick and thin films to trichome‐bearing surfaces, including the physical requirements of the thin‐film electrode. Trichomes hinder the thick film from conforming to the surface, whereas the thin film exhibits lower flexural rigidity and is partially pierced by trichomes. SWCNTs are deposited onto ultrathin SBS films, yielding a transparent, water‑vapor‑permeable, water‑resistant, non‑invasive electrode that conforms to complex leaf surfaces. (b) Photographs of SWCNT nanofilm electrodes attached to different leaves: on a soybean leaf (left; 70 nm‐thick electrode) and on a pothos leaf (right; 130 nm‐thick electrode). In the soybean case, the electrode is pierced by trichomes, showing conformal adhesion to the “hairy” surface. The electrodes cover the areas outlined by the red dashed lines. (c) SEM image of a SWCNT nanofilm at 40 000× magnification. (d) UV–vis transmittance spectra of 480 nm SWCNT nanofilm and commercial hydrogel electrodes (i.e., transparent hydrogel electrodes and opaque hydrogel electrodes). Each sample was deposited on quartz substrates. (e) Schematic illustration of the measurement setup, showing the attachment positions of the recording and reference electrodes, as well as the area covered by Al foil. (f) A typical LIB waveform measured using hydrogel electrodes on a pothos leaf, displaying an initial hyperpolarization phase (P1), followed by a depolarization phase (P2). LIB amplitude (Δ*V*) is defined as the difference between P1 and P2.

Trichomes, the hair‐like structures on the epidermis of many plants, play important roles in plant growth and are widely observed in economically important crops such as Solanaceae and legumes [1, 2, 3, 4]. Although monitoring physiological signals in trichome‐bearing plants is an urgent task for global food security [5, 6, 7, 8, 9], the design and selection of electrodes suitable for trichome‐bearing surfaces remains underexplored. We propose that such electrodes must be transparent to avoid inhibiting photosynthesis, water‐resistant against rain, non‐invasive to plant tissues, and conformable to the complex trichome‐bearing surface (Figure 1a). Trichomes serve key physiological functions (e.g., regulating transpiration, facilitating gas exchange, ion homeostasis, and enabling secretion activity [2, 4, 10]); thus, electrodes must not completely cover or mechanically crush them.

Previous approaches to plant electrophysiology have employed needle‐like electrodes [11, 12, 13, 14, 15], hydrogel‐based electrodes [9, 16, 17, 18, 19], metal‐based thin electrodes (e.g., gold patterns on polyethylene terephthalate (PET) substrates and stand‐alone nickel patterns) [20, 21], and flexible films (e.g., silver nanowires and poly(3,4‐ethylenedioxythiophene):poly(4‐styrenesulfonate) (PEDOT:PSS)) [22, 23, 24, 25]. Needle electrodes can acquire high‐quality electrical signals by bypassing trichomes and inserting directly into the tissue. However, this insertion caused physical damage, which not only introduces damage‐induced signals requiring hours for acclimation [26, 27], but also creates wound sites that facilitate pathogen entry [28], potentially compromising plant health [14, 29]. Consequently, hydrogel‐based ionotronic interfaces have gained attention as non‐invasive electrodes. Recent advances in ionotronic interfaces, such as morphable thermogel electrodes [9, 19], have achieved remarkable conformability to trichome‐bearing surfaces and low interfacial impedance. However, a critical limitation of these systems in open‐field agriculture is their environmental instability; hydrogel electrodes intrinsically lack water resistance because they typically rely on hydrophilic polymers. Some papers also reported that hydrogel electrodes are invasive, making them unsuitable for long‐term monitoring [22, 30]. Furthermore, hydrogel electrodes inhibit physiological activities such as transpiration because they completely cover the leaf surface, including trichomes.

Dry electrodes, which do not require adhesives, appear to be more useful for outdoor measurements. However, conventional metal‐based electrodes generally suffer from poor conformability to rough surfaces, especially trichome‐bearing surfaces, resulting in high interfacial impedance [24, 31, 32, 33]. Conversely, nanoscale thin‐film electronics have enabled conformal adhesion to uneven biological surfaces, and recent reports suggest that flexible electrodes can adapt to trichome‐bearing leaves [21, 23, 24]. Nonetheless, no systematic studies have reported how the electrode's physical properties, especially thickness, influence conformability and signal quality in trichome‐rich plants.

In this study, we addressed these limitations by developing thin‐film polymer composite electrodes that are pierceable by trichomes and integrate seamlessly with the leaf surfaces of diverse species. Hydrophobic single‐walled carbon nanotubes (SWCNTs) were employed as a conductive network material, and poly(styrene‐*b*‐butadiene‐*b*‐styrene) (SBS) was adopted as a supporting layer. SWCNT bilayer nanofilms were fabricated by gravure coating, and we systematically varied the overall film thickness to determine its role in achieving conformability on trichomecovered surfaces (Figure 1b). We found that thicknesses of 70–320 nm enable effective electrode–leaf adhesion. Notably, 70 nm‐thick films exhibited robust conformability, which implies minimal sensitivity to variations in the trichome density, stiffness, and shape. This represents the first demonstration of nanoscale conductive films that can achieve conformal contact with trichomes through the controlled design of their thickness. We further evaluated the long‐term non‐invasiveness and water resistance of the SWCNT nanofilms electrodes on leaf surfaces, comparing their performance with that of PEDOT:PSS nanofilms and commercial pre‐gelled Ag/AgCl electrodes. Finally, we demonstrated that the proposed electrodes can detect chemical stress by recording light‐induced biopotential (LIB) in leaves treated with the photosynthesis inhibitor 3‐(3,4‐dichlorophenyl)‐1,1‐dimethylurea (DCMU), indicating their feasibility for monitoring plant physiology in smart agriculture systems. Ultimately, our pierceable, water‐resistant, and transparent SWCNT nanofilm electrodes can be used in real‐time monitoring systems to improve agricultural efficiency.

## 2 Results and Discussion

### 2.1 Characterization of the SWCNT Nanofilms

SWCNT nanofilm composites were fabricated by coating an SWCNT aqueous dispersion onto an SBS layer using the gravure coating method (Figure S1). In this composite design, the SBS layer functions as a highly stretchable, self‐supporting elastomeric substrate, and the SWCNT layer forms a conductive fiber network that can deform with the substrate. SWCNTs are anchored to the hydrophobic SBS surface primarily through physical adsorption, without the use of chemical adhesives. This binder‐free composite design enables SWCNT nanofilm electrodes to retain both the intrinsic flexibility of the elastomer and the high conductivity of the SWCNT network. Figure 1c shows a scanning electron microscopy (SEM) image of the as‐prepared SWCNT nanofilm surface, exhibiting a fiber network structure comprising SWCNT bundles, which has been characterized and discussed in our previous paper [34]. The thicknesses of the SWCNT nanofilms were controlled to 70, 320, and 480 nm, as detailed in Figure S2. Controlling the films to nanometer‐scale thicknesses ensured high transparency comparable to recent high‐performance plant surface electrodes, with more than 80% light transmittance for wavelengths of 400–1100 nm (Figure 1d; Table S1). In contrast, conventional transparent hydrogel electrodes transmitted only ∼40% of the light, and opaque hydrogel electrodes were not light‐transmissive (< 1%) owing to the use of non‐woven fabrics and translucent film as the base material (Figure 1d), which would inhibit photosynthesis. The fiber network structure of SWCNT and the micro‐Brownian motion characteristics of the molecular chains of polybutadiene in SBS nanofilms provide a high water vapor transmission rate (i.e., 28,316 g m^−2^(2h)^−1^) [34], which is necessary for allowing plant transpiration. Moreover, impedance measurements were conducted for each electrode on a pothos leaf to confirm the contact impedance, showing that the impedance magnitude and phase angle of SWCNT nanofilms were comparable to those of PEDOT:PSS nanofilms for all frequencies (Figure S3).

### 2.2 Conformability to Trichome‐Bearing Leaves

The key issue associated with the practical application of nanofilm electrodes as plant sensors is attaching them to uneven surfaces with trichomes, which are found on the leaves of many vegetables. Herein, we demonstrated the attachment of SWCNT nanofilm electrodes on trichome‐rich soybean leaves. For the thin film to adhere to uneven surfaces with trichomes, the film's flexural rigidity must be sufficiently low. The following equation defines the flexural rigidity:

(1) $$\begin{array}{c} \;D=\frac{Ed_{0}^{3}}{12\left(1-\nu^{2}\right)}\; \end{array}$$

where *D*, *E*, *d_0_*, and *ν* denote the flexural rigidity, Young's modulus, film thickness, and Poisson's ratio, respectively. Given that flexural rigidity is proportional to the cube of the film's thickness, the most effective strategy to reduce flexural rigidity is to make the film thinner. Therefore, SWCNT nanofilm electrodes were prepared using 5, 2.5, and 1 wt.% SBS solutions, resulting in bilayer film thicknesses of 480, 320, and 70 nm, respectively. These electrodes were applied to soybean leaves to evaluate their adhesion.

To demonstrate the signal acquisition using the SWCNT nanofilm electrodes, we measured the LIB of soybean and pothos leaves using recording and reference electrodes (Figure 1e,f). The LIB response reflects extracellular potential changes mediated by ions (e.g., H^+^, Ca^2^^+^, K^+^, and Cl^−^) under light stimulation [16], and the LIB waveform varies with the incident light wavelength [11]. temperature [35], ambient gas composition [36], and salt stress [16, 37]. Figure 1f shows a typical waveform of the LIB response, measured using hydrogel electrodes under our measurement system; an initial hyperpolarization phase (P1) lasting 1–2 min occurs immediately after light irradiation, followed by a depolarization phase (P2) lasting 10–20 min and subsequent repolarization toward the resting potential. This is consistent with previous studies [11, 16, 36].

**FIGURE 2:**
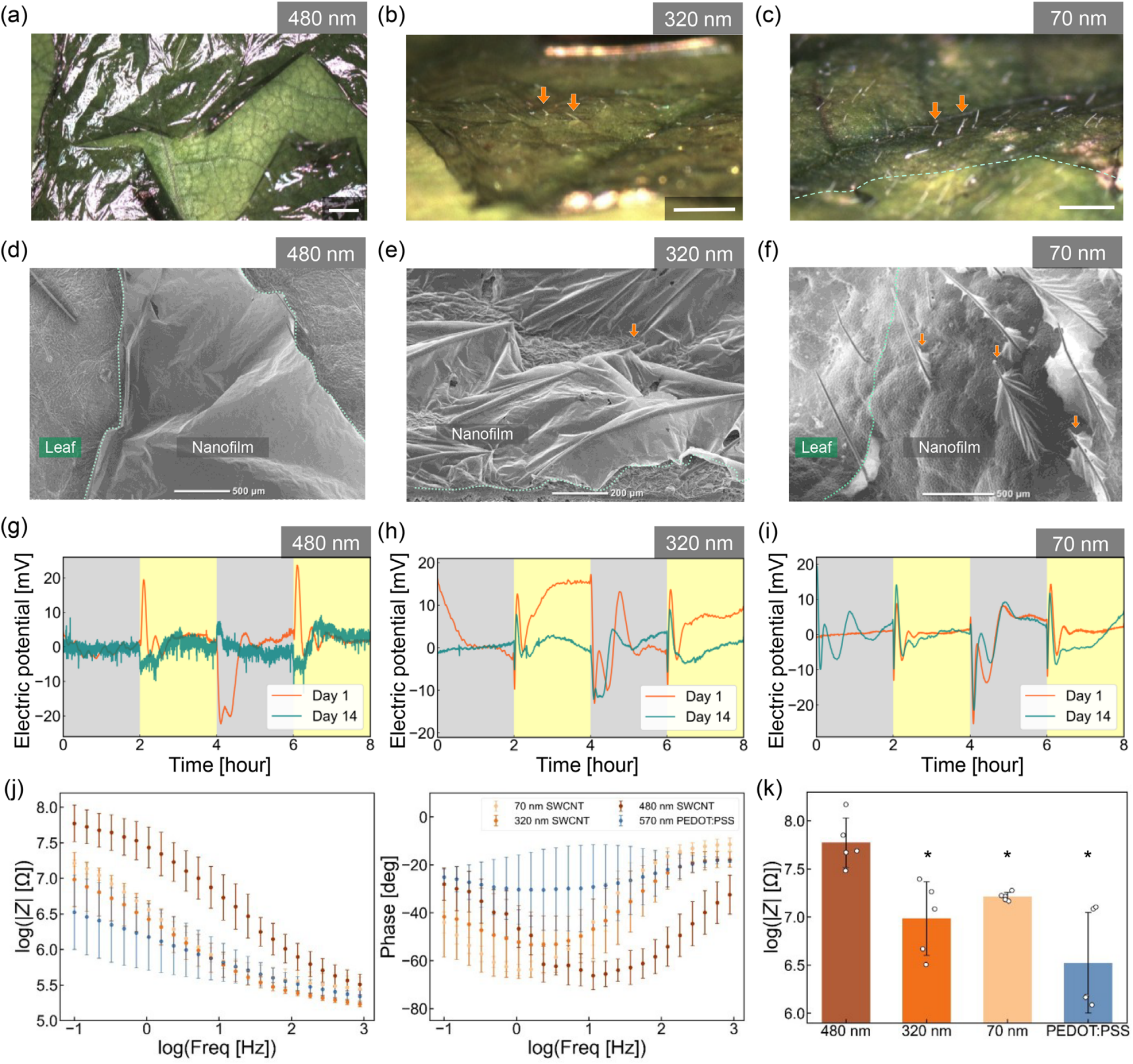
Conformability of SWCNT nanofilms to trichome‐bearing surfaces. (a–c) Stereomicroscopic images of SWCNT nanofilm electrodes with thicknesses of 480 nm (a), 320 nm (b), and 70 nm (c). Scale bars indicate 1 mm. Arrows indicate trichomes piercing the films. The green dashed line indicates the border between the film and the leaf. (d–f) SEM images of SWCNT nanofilm electrodes with thicknesses of 480 nm (d), 320 nm (e), and 70 nm (f). Arrows highlight the regions pierced by trichomes. In the 320 nm film (e), the tips of some trichomes pierce the film and portions of the film are attached to the leaf surface, whereas in the 70 nm film (f), localized tearing by trichomes allows the remaining sheet to conform more extensively and stably to the leaf surface. The green dashed lines indicate the border between the film and the leaf. The 70 nm film (the right region) conforms so tightly to the epidermal tissue that its presence is almost indistinguishable. (g–i) LIB measurement on Days 1 and 14 using SWCNT nanofilm electrodes with thicknesses of 480 nm (g), 320 nm (h), and 70 nm (i). (j,k) Bode plot showing the impedance magnitudes and phase responses of 70–480 nm SWCNT nanofilms and 570 nm PEDOT:PSS nanofilms attached to soybean leaves (j). Bar graphs comparing the impedance of 480, 320, and 70 nm SWCNT nanofilms, as well as PEDOT:PSS nanofilms, at 0.1 Hz (k). The 70 and 320 nm SWCNT nanofilms, as well as PEDOT:PSS nanofilms, exhibit effective nanofilm‐leaf contact compared with 480 nm SWCNT nanofilms. Values are the means ± standard deviation of five independent experiments. ^*^ *p* < 0.05, one‐way analysis of variance with Tukey's test in 480 nm SWCNT nanofilms versus 70–320 nm SWCNT nanofilms and PEDOT:PSS nanofilms. Statistical analyses were performed on raw data before logarithmic transformation.

We first evaluated the attachment of SWCNT nanofilms to soybean leaves. Figure 2a–c shows stereomicroscopic images of SWCNT nanofilms with thicknesses of 480, 320, and 70 nm, applied to soybean leaves. The 480 nm films appeared to float on the leaf surface because of the trichomes, and the films were broken after drying (Figure 2a). SEM images also showed that the 480 nm films were inhibited by the trichomes and floated away from the leaf surface (Figure 2d). The 320 nm films adhered more closely to the leaf surface, but some areas were floating (Figure 2b). This suggested that the tips of some trichomes pierced the film, allowing some areas to adhere well (Figure 2e; Figure S4a). Notably, the 70 nm films adhered closely and uniformly to the leaf surface. Stereomicroscopic images (Figure 2c) and SEM images (Figure 2f; Figure S4b) revealed that trichomes pierced the films, and we attributed the adhesion of the thin films to this trichome‐piercing phenomenon on the trichome‐rich surface. Moreover, the SEM images revealed regions where the 70 nm SWCNT nanofilm closely conformed and integrated with the epidermal tissue structure.

To validate the universality of this trichome‐piercing mechanism, we evaluated the conformability of SWCNT nanofilms on pumpkin and eggplant leaves possessing surface morphologies distinct from those of soybean leaves. Consistent with our findings on soybean leaves, conformability improved significantly with reduced film thickness (Figures S5 and S6). While thicker films (400–480 nm) were lifted by trichomes and failed to contact the leaf surface, thinner films (70–320 nm) exhibited the trichome‐piercing phenomenon, enabling conformal contact with the leaf surface. Notably, the 70 nm SWCNT nanofilms established an intimate biointerface with the intricate vertical and horizontal trichomes of eggplant leaves (Figure S6c). This confirms that the reduced flexural rigidity of the ultra‐thin film enables conformal adaptation to highly complex surface morphology.

LIB measurements were recorded immediately after electrode application (Day 1) and after 2 weeks of attachment (Day 14). Figure 2g–i compares the LIB responses obtained on Days 1 and 14 using SWCNT nanofilm electrodes of each thickness. For the 480 nm films, LIB waveforms could be measured immediately after application. However, after 14 days, the film was torn by the trichomes, and LIB measurements could no longer be taken (Figure 2g). In contrast, the 320 and 70 nm films provided accurate LIB measurements even on Day 14, as on Day 1 (Figure 2h,i).

Then, impedance measurements were conducted for 70, 320, and 480 nm SWCNT nanofilms, as well as a 570 nm PEDOT:PSS nanofilm, whose thickness is comparable to that of previously reported PEDOT:PSS films (400–600 nm) [22, 23, 38], (Figure 2j,k). At 0.1 Hz, the 70 nm SWCNT nanofilm, 320 nm SWCNT nanofilm, and PEDOT:PSS nanofilms exhibited impedances of 1.6 × 10^7^, 1.2 × 10^7^, and 5.8 × 10^6^ Ω, respectively. In contrast, the 480 nm SWCNT nanofilm presented a significantly higher impedance of 8.1 × 10^7^ Ω (Figure 2k), which was attributed to poor conformability and the formation of air gaps between the film and leaf surfaces. Phase profiles were comparable across all samples (Figure S7). The relatively large errors observed for the 320 nm SWCNT nanofilm, 480 nm SWCNT nanofilm, and PEDOT:PSS nanofilm samples suggest the unstable and non‐uniform contact caused by floating areas, which strongly depends on variations in trichome density or other surface features. Notably, 70 nm SWCNT nanofilms showed smaller error bars, implying that their conformability is less sensitive to such variations through universal trichome‐piercing. However, the 70 nm SWCNT nanofilms occasionally exhibited slightly higher impedance than the 320 nm films, likely due to small tears induced by trichomes, highlighting the importance of optimizing film thickness for stable and reproducible performance.

These results suggest that the trichome‐piercing morphology of the thinner SWCNT nanofilms (70–320 nm) enhances conformability by mitigating the physical hindrance of trichomes, whereas the thicker 480 nm films are too difficult to puncture and become inhibited by trichomes, leading to unstable, floating contact that results in both higher impedance and larger sample‐to‐sample fluctuations. Conversely, the 570 nm PEDOT:PSS nanofilms maintained low impedance despite their relatively high thickness, likely because PEDOT:PSS swells and softens with water when it is transferred to the leaf surface, allowing the film to envelop trichomes and adhere tightly to the leaf surface (Figure S4c). Overall, these findings demonstrate that reducing the film thickness effectively suppresses trichome interference and that water‐resistant SWCNT nanofilms can achieve contact impedances comparable to those of conventional PEDOT:PSS films. Moreover, stable biopotential measurements were maintained after 2 weeks of continuous attachment using the 70 and 320 nm SWCNT nanofilm electrodes.

### 2.3 Non‐Invasiveness in Long‐Term Measurements

**FIGURE 3:**
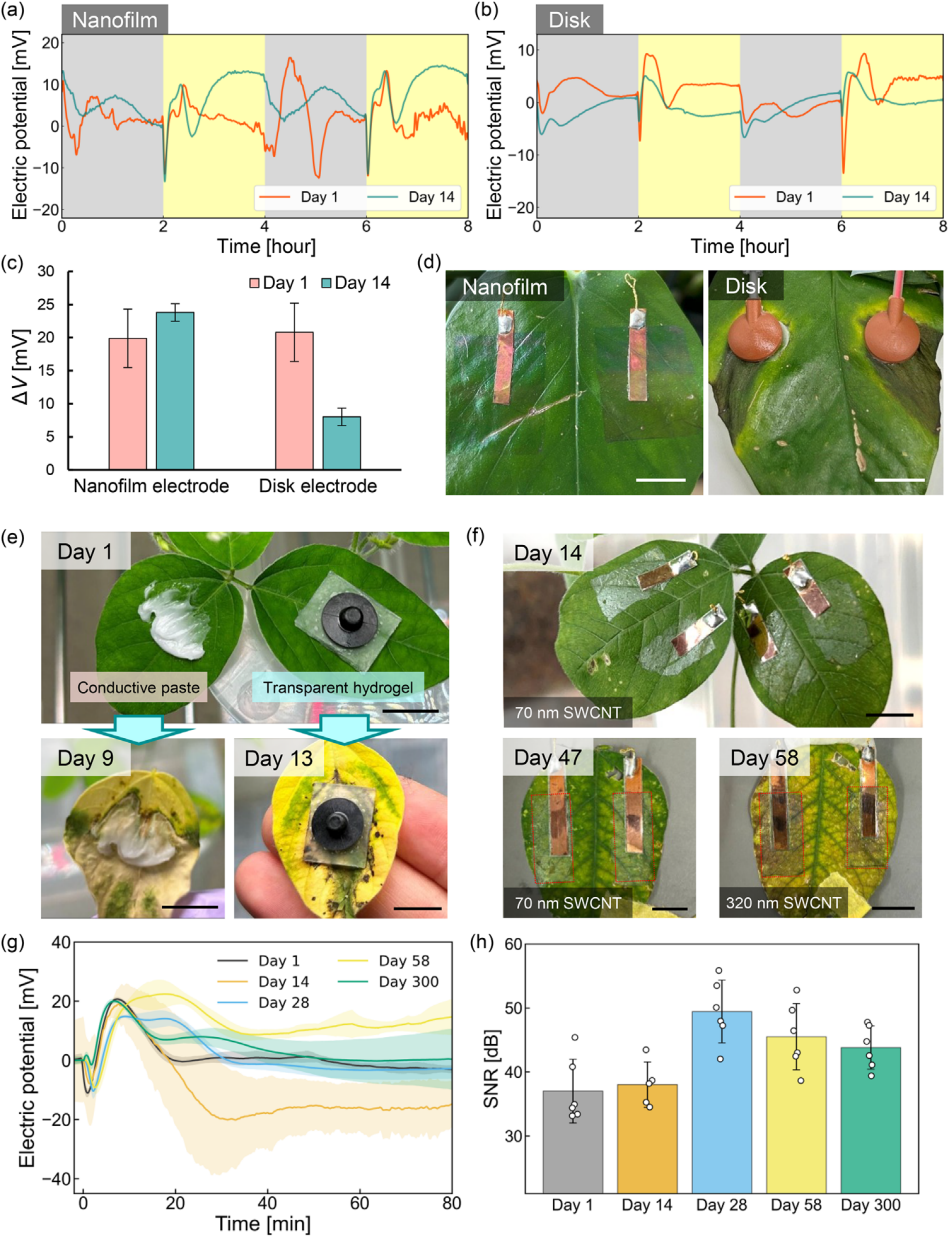
Non‐invasiveness of long‐term measurements compared with commercial electrodes. (a,b) Representative LIB responses of 480 nm SWCNT nanofilm electrodes (a) and commercial disk electrodes (b) during two cycles of illumination and dark periods, extracted from the full day of measurements (i.e., six cycles). (c) Comparison of LIB amplitudes between SWCNT nanofilm electrodes and disk electrodes on Days 1 and 14. Error bars denote the standard deviation. (d) Images of a pothos leaf after attachment of SWCNT nanofilm electrodes (left) and disk electrodes (right) for 2 weeks. The leaf tissue around the attachment area of the disk electrodes showed color changes, indicating invasiveness. SWCNT nanofilm electrodes exhibited non‐invasiveness. Scale bars indicate 1 cm. (e,f) Pictures of soybean leaves after attachment of transparent hydrogel electrodes and conductive paste for disk electrodes (e), as well as SWCNT nanofilm electrodes (f). The attachment area of the hydrogel and disk electrodes showed invasiveness within 2 weeks, whereas all the SWCNT nanofilm electrodes showed non‐invasiveness even after 2 months. Scale bars indicate 1 cm. The electrodes cover the areas outlined by the red dashed lines. (g,h) Long‐term stability of LIB signals recorded from a pothos leaf using attached SWCNT nanofilm electrodes (130 nm‐thick electrodes) over a period of 10 months. Distinct LIB waveforms were successfully acquired even after 300 days of attachment (g). The SNR remained comparable from Day 1 to Day 300, indicating that high signal quality was preserved (h). SNR was calculated based on the amplitude of each LIB waveform (n = 6 for each day, except for Day 14, where n = 5 due to a temporary suspension of the data logger). Error bars denote standard deviation.

To evaluate the long‐term performance and non‐invasiveness, SWCNT nanofilm electrodes (480 nm) and disk electrodes were affixed to pothos leaves for a period of 2 weeks, and the LIB changes were continually monitored on Days 1 and 14 (Figure 3a,b). The LIB amplitudes recorded using the SWCNT nanofilm electrode on Day 14 were comparable to those observed on Day 1, whereas the disk electrodes showed a marked reduction in amplitude on Day 14, approximately one‐third of the amplitude observed on Day 1 (Figure 3c). Visual inspection on Day 14 further revealed that leaves attached to SWCNT nanofilm electrodes exhibited no discoloration, whereas those with disk electrodes developed noticeable blackening at the attachment area (Figure 3d). This harmful effect is likely due to the presence of NaCl in the conductive paste used for attachment. Sodium ions in conductive pastes can penetrate the epidermal tissue and induce cellular dehydration through osmotic pressure over prolonged periods of attachment [30]. We also evaluated the hydrogel electrodes, which contain sodium ions in the conductive hydrogel; harmful effects were not observed even after 14 days, and the LIB waveforms on Day 14 were similar to those obtained on Day 1 because of the thick cuticle of pothos leaves (Figure S8).

Next, we attached SWCNT nanofilm electrodes, hydrogel electrodes, and disk electrodes to trichome‐rich leaves (i.e., soybean and tobacco leaves) for over 14 days and monitored their effects (Figure 3e,f; Figure S9). On both leaves, sites covered with the opaque hydrogel electrodes, transparent hydrogel electrodes, and conductive paste from the disk electrodes began to show discoloration within 1 week of application, and by Day 14, this discoloration had spread to the surrounding tissue (Figure 3e; Figure S9). In contrast, the attachment areas of the SWCNT nanofilm electrodes remained undamaged throughout the entire period, and we confirmed minimal invasiveness over several months of attachment (Figure 3f; Figure S9). Unlike pothos leaves, the soybean and tobacco leaves were significantly damaged by all of the wet electrodes, making them unsuitable for long‐term, non‐invasive monitoring.

To demonstrate the exceptional long‐term measurement capability of the SWCNT nanofilm electrodes, we monitored LIB signals typically for 1–2 months, with a maximum duration extending to 10 months (Figure 3g; Figures S10 and S11). Distinct LIB waveforms were successfully acquired on all measurement days throughout these 10 months (Figure 3g). Furthermore, the signal‐to‐noise ratio (SNR) remained comparable from Day 1 to Day 300, confirming that high signal quality was preserved for the entire duration (Figure 3h).

Although most nanofilm electrodes exhibited high signal quality even after 1 and 2 months, in some instances, the recordings became noisier over time (Figures S10 and S11). This increased noise was likely caused by cracking at the interface between the SWCNT nanofilm and the interconnects, namely the Cu‐based collecting electrodes, due to leaf vibrations. However, this noise did not significantly affect the measurement capability; clear LIB waveforms could be successfully obtained by applying signal processing (e.g., Savitzky‐Golay filter) (Figure S11c). These results highlight the superior long‐term performance of SWCNT nanofilm electrodes compared with conventional electrodes and underscore their potential for durable, non‐invasive plant monitoring (Table S1).

### 2.4 Durability Against Water Flow

**FIGURE 4:**
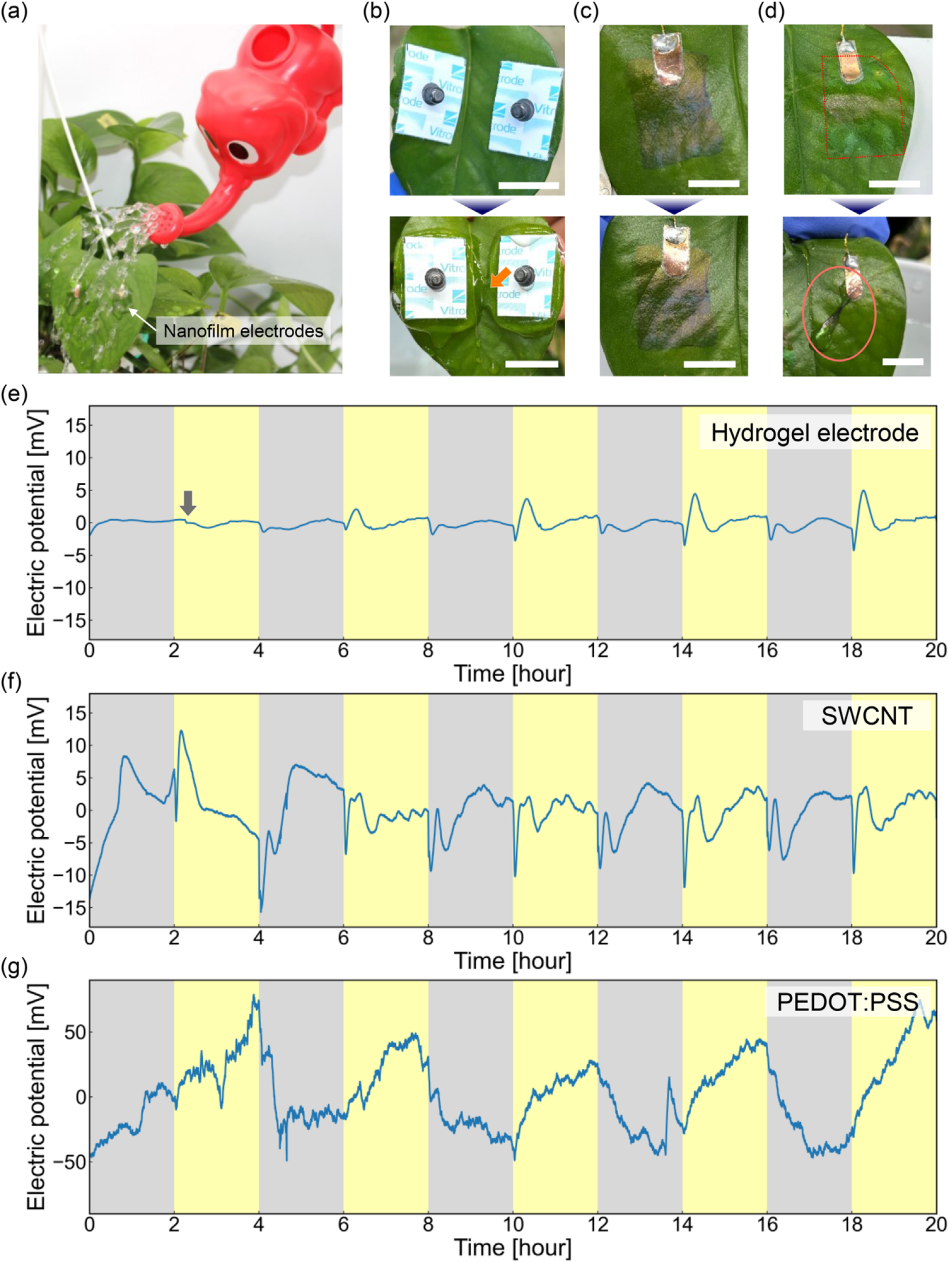
Water resistance of the SWCNT nanofilm electrodes. (a) A photograph of simulated rainfall applied to the leaf surfaces. (b–d) Photographs of a hydrogel electrode (b), SWCNT nanofilm electrode (c), and PEDOT:PSS nanofilm electrode (d) before and after the exposure to simulated rain for 30 s. The arrow indicates contact between the recording and reference hydrogel electrodes. The PEDOT:PSS nanofilm electrode covers the area outlined by the red dashed line. The red circle indicates the detached PEDOT:PSS nanofilm electrode. Scale bars indicate 1 cm. (e–g) LIB measurements following the water flow test using the hydrogel electrodes (e), SWCNT nanofilm electrodes (f), and PEDOT:PSS nanofilm electrodes (g). The arrow indicates the failure of the LIB measurement in the first irradiation cycle using the hydrogel electrodes.

To evaluate the practicality of using the electrodes for outdoor applications, their performance on pothos leaves was evaluated under simulated heavy rainfall (vigorously spraying a total of ∼1000 mL of water over 30 s) (Figure 4a; Movies S1–S3). Figure 4b–d shows the hydrogel, SWCNT nanofilm, and PEDOT:PSS nanofilm electrodes before and after exposure to strong water flow for approximately 30 s. Water caused the hydrogel electrode to swell, resulting in a short circuit between the measurement and reference electrodes (Figure 4b; Movie S1). Therefore, the LIB signal could not be detected with hydrogel electrodes during the first 2 h following water exposure. After 6 h, the LIB signal became measurable again, suggesting that the water absorbed by the hydrogel electrode had evaporated and alleviated the short circuit (Figure 4e). Figure 4c,d, and Movies S2 and S3 show that SWCNT nanofilm electrodes had high durability against running water, whereas PEDOT:PSS nanofilm electrodes exhibited detachment. Figure 4f,g shows the LIB signals acquired using SWCNT nanofilm and PEDOT:PSS nanofilm electrodes after water exposure. The SWCNT nanofilm signal drifted by approximately 100 mV, exhibiting a larger drift than the hydrogel electrode (Figure S12), but the LIB waveform could still be acquired (Figure 4f). In contrast, the PEDOT:PSS nanofilm electrode failed to acquire meaningful LIB waveforms owing to delamination (Figure 4g).

In addition to the simulated rainfall test, we conducted a 30‐min immersion test to evaluate the effects of severe, prolonged water exposure, such as continuous heavy rain. We compared the LIB signals and impedance of the hydrogel, PEDOT:PSS nanofilm, and SWCNT nanofilm electrodes before and after immersion (Figures S13–S15). For the hydrogel electrodes, the swollen hydrogel caused a short circuit between the measurement and reference electrodes, resulting in a complete failure in the LIB measurement right after immersion. LIB signals could not be acquired until the swollen hydrogel had gradually dried, consistent with the results of the simulated rainfall test (Figure S13). Impedance measurements showed that the impedance magnitude of the hydrogel electrode at 0.1 Hz decreased significantly from 1.3 MΩ to 480 kΩ, indicating instability in electrical performance due to gel swelling. The phase response between 1–100 Hz also exhibited distinct changes (Figure S15). In contrast, clear LIB signals were successfully recorded using the PEDOT:PSS and SWCNT nanofilms (Figure S14), and both electrodes exhibited comparable impedance spectra before and after immersion (Figure S15). This stability indicates that the ultra‐thin nanofilms intimately adhere to the leaf surface, effectively preventing water ingress at the interface and thereby maintaining a constant contact impedance.

Hydrogel electrodes strongly adhere to the leaf surface via hydrogen‐bonding networks within the gel, allowing them to maintain contact with the leaf surface during swelling, although the adhesion weakens and they can easily be peeled off. However, the adhesion of dry electrodes, such as SWCNT and PEDOT:PSS nanofilms, depends on weaker van der Waals interactions [23]. Because PEDOT:PSS is hydrophilic, exposure to water causes swelling and the accumulation of water at the leaf–nanofilm interface, disrupting the weak interfacial interactions and inducing delamination under dynamic water flow. Conversely, SWCNTs are hydrophobic, preventing water ingress at the interface and thus maintaining conformal contact even under both static immersion and dynamic water flow. Compared with recently reported plant electrodes (Table S1), this robust dual‐mode water resistance is a distinct advantage of our system, ensuring highly reliable durability for practical outdoor measurements. Consequently, SWCNT nanofilm electrodes demonstrated superior water resistance compared with conventional electrodes under heavy rain conditions.

### 2.5 Photosynthesis Inhibition

**FIGURE 5:**
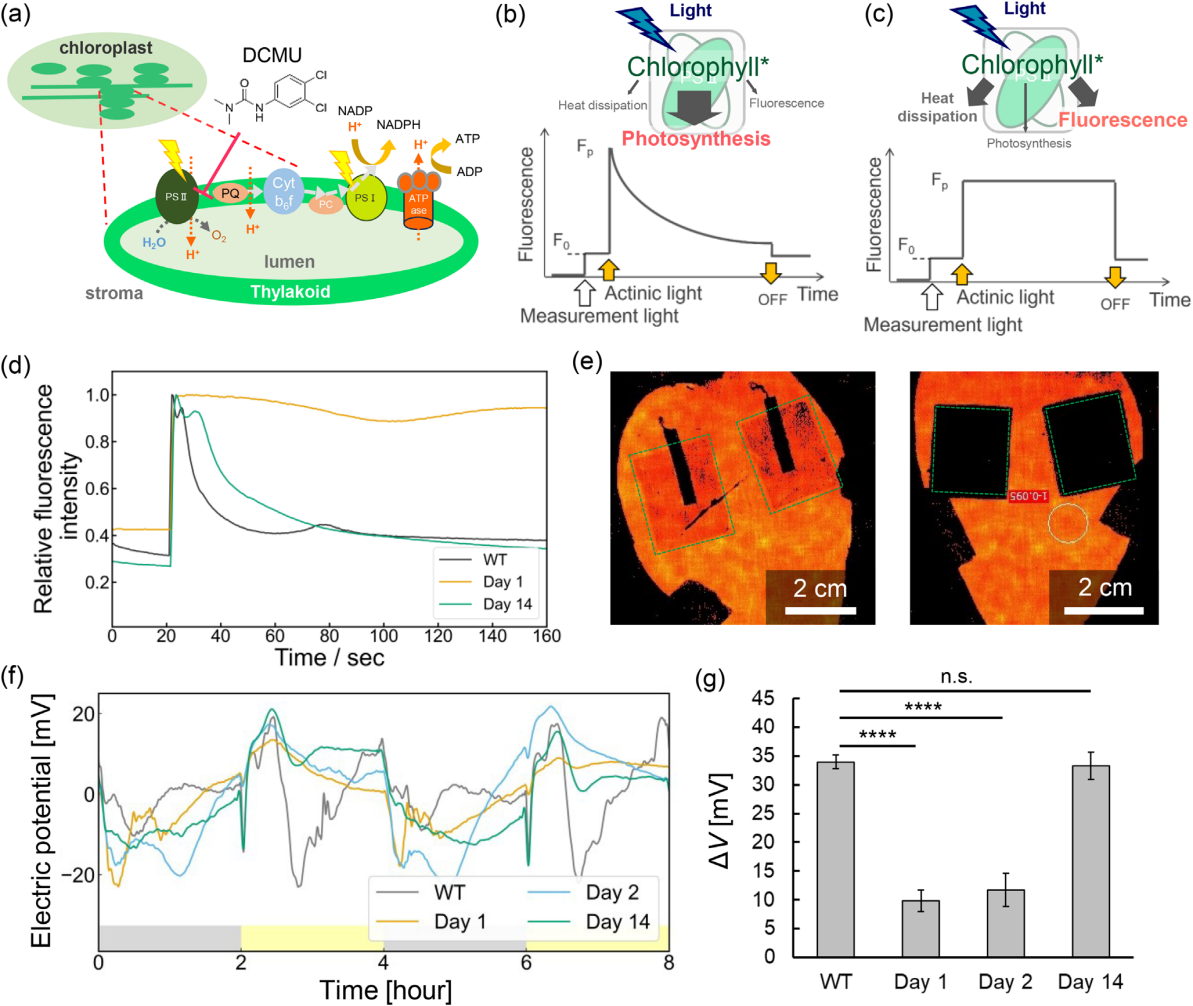
Application of the SWCNT nanofilm electrode for detecting photosynthesis inhibition. (a) A schematic illustration of photosynthesis inhibition by DCMU. (b,c) Chlorophyll fluorescence induction kinetics in healthy leaves (b) and in DCMU‐treated leaves (c). In WT leaves, the chlorophyll fluorescence decreases from the momentary maximum level (F_p_) after actinic light illumination (b). In DCMU‑treated leaves, the chlorophyll fluorescence remains high at F_p_ and does not decrease. (d) Relative fluorescence intensity of leaves before treatment (WT), immediately after treatment (Day 1), and 14 days after treatment (Day 14). Chlorophyll fluorescence was measured under an actinic light of 80 µmol/m^2^s. (e) 2D‐PAM fluorescence images of a leaf with the SWCNT nanofilm electrodes attached (left) versus a leaf with hydrogel electrodes (right). Green dashed lines indicate the electrode attachment areas. (f, g) Representative LIB traces recorded for WT and DCMU‐treated leaves on Days 1, 2, and 14 (f) and the LIB amplitude for each day (g). Values are the means ± standard deviation of five measurements per day under daily cycles of 2 h illumination/2 h darkness. The Student's t‐test was used to compare two groups. ^****^ *p* < 0.0001; ns, no significant difference.

Based on the performance evaluations detailed in the previous chapters, we demonstrated the application of our SWCNT nanofilm electrode for detecting photosynthesis inhibition. We employed DCMU, a widely used herbicide and photosynthesis inhibitor, to inhibit electron transport in plant cells. DCMU binds to Photosystem II (PSII) on the thylakoid membranes of chloroplasts, thereby blocking electron transport (Figure 5a). As a result, light‐induced changes in intracellular membrane potential are suppressed in DCMU‐treated cells [39]. Consequently, we aimed to detect the reduction in the LIB amplitude using SWCNT nanofilm electrodes.

The induction of chlorophyll fluorescence (i.e., the Kautsky effect) can be measured by pulse‐amplitude‐modulated (PAM) fluorometry to confirm the inhibition effect of DCMU on photosynthesis [40, 41]. Before conducting the measurement, it is necessary to keep the leaves in the dark for over 30 min to oxidize the photosynthetic electron transfer chain. Upon illumination with actinic light, chlorophyll fluorescence increases to the temporary maximum level (F_p_) as a result of quinone A (Q_A_) reduction within PSII, and in healthy leaves, this fluorescence gradually decreases owing to the oxidation of Q_A_ through photosynthetic electron transport (Figure 5b). In contrast, under conditions where photosynthetic electron transport is inhibited by DCMU, the oxidation of Q_A_ is suppressed, and chlorophyll fluorescence is expected to remain at a high level (Figure 5c). Therefore, by comparing the induction of chlorophyll fluorescence, the effect of DCMU can be evaluated.

Time‐course measurements of chlorophyll fluorescence in DCMU‐treated leaves confirmed the inhibition of PSII oxidation on Day 1. However, by Day 14, the inhibitory effect appeared to have dissipated (Figure 5d), indicating that DCMU is metabolized and detoxified within 2 weeks in pothos leaves [42, 43]. We also confirmed that, unlike the hydrogel electrodes, the transparency of SWCNT nanofilm electrodes enabled chlorophyll fluorescence measurements to be performed while the electrode remained attached to the leaf (Figure 5e). Our SWCNT nanofilm electrodes demonstrated a significant reduction in LIB amplitude immediately following DCMU treatment (Days 1 and 2), with the amplitude on Day 1 being roughly one‐third that of the wild‐type (WT) leaf measured before treatment (Figure 5f,g). By Day 14, when the inhibition had subsided, no significant difference was observed compared with the WT sample (Figure 5g). Notably, hydrogel electrodes showed similar results to the SWCNT nanofilm electrodes (Figure S16). In the control test, using the control solution without DCMU, the decrease in chlorophyll fluorescence after illumination confirmed that the electron transport chain was functioning properly and that photosynthesis was not inhibited (Figure S17a). Then, the LIB amplitude exhibited a significant reduction on Days 1 and 2, with the amplitude on Day 1 being approximately half that of WT leaves (Figure S17b,c). By Day 3, the LIB amplitude had recovered to WT levels. These results suggest that the control solution, which only contains dimethyl sulfoxide (DMSO), initially affects the physiological conditions of the leaf, but its inhibitory effect disappears within approximately 2 days.

These findings indicate that, compared with the control, DCMU treatment leads to a marked reduction in LIB amplitude, suggesting that the suppression of LIB is attributable to the inhibition of PSII by DCMU. The mechanism for the decrease in LIB amplitude can be described as follows. In chloroplasts, the electron transport chain during photosynthesis drives ATPase, which facilitates the movement of H^+^, leading to changes in ion concentrations between the thylakoid lumen and the stroma [39, 44]. Changes in the stromal ion concentration affect intracellular ion levels, and the activation of ion channels in the plasma membrane subsequently alters the extracellular ion concentration. In DCMU‐treated leaves, blocking electron transport prevents proton flux and the associated changes in stromal and extracellular ion concentrations, which explains the observed suppression of LIB amplitude [39]. Moreover, we observed the detoxification of DCMU through the recovery of the LIB amplitude. These results demonstrate that the inhibitory effects on photosynthesis can be evaluated by measuring LIB in a manner comparable to chlorophyll fluorescence analysis. Therefore, this approach holds potential for the quantitative assessment of herbicide damage.

## 3 Conclusion

We developed SWCNT nanofilm electrodes that overcome key limitations of conventional plant surface sensors, providing a non‐invasive, water‐resistant, and pierceable platform for long‐term biopotential monitoring on trichome‐rich surfaces. By varying the thickness, we found that 70–320 nm films exhibited a unique and critical advantage: enhanced conformability to trichome‐rich leaf surfaces via a universal trichome‐piercing mechanism. In contrast to recent high‐performance electrodes that often face trade‐offs between conformability and environmental durability, our nanofilms simultaneously achieved high transparency (> 80%), high water vapor permeability, robust water resistance against rain, and long‐term stability (> 2 months) without sacrificing adhesion to complex leaf surfaces. Consequently, this is the first report to improve the conformability of water‐resistant, transparent, and non‐invasive dry electrodes by leveraging ultrathin electrodes with a minimum thickness of 70 nm. Notably, the 70 nm‐thick film achieved conformal contact even on complex trichome structures, demonstrating universal conformability through a trichome‐piercing mechanism. The transparency of the proposed electrodes also allows simultaneous chlorophyll fluorescence imaging, and their hydrophobicity ensures reliable performance under simulated rain conditions. We validated the ability to detect photosynthesis inhibition via DCMU‐induced changes in the LIB amplitude, which indicate reductions in electron transport. While our results demonstrate robust and long‐term biopotential sensing, we recognize opportunities to enhance performance further. In particular, integrating a semi‐dry or adhesive conductor without irritating leaf tissues would combine the low impedance and conformable interface with the durability and water resistance of dry films [33, 45, 46]. Further investigation is also necessary to improve the electrode interconnects, providing more stable measurements against vibration and rain. Moreover, machine learning approaches can transform raw LIB signals into early, quantitative indicators of plant stress and disease. This work establishes our pierceable SWCNT nanofilm electrode platform for reliable, long‐term plant biopotential monitoring, paving the way for practical, data‐driven smart agriculture.

## 4 Experimental Section

### 4.1 Materials

Polyvinyl alcohol (PVA) (Mw 22 000, 86.5%–89% hydrolyzed) was purchased from Kanto Chemical Co., Inc., Tokyo, Japan. SBS (Mw 140 000) was purchased from Sigma‐Aldrich Co. LLC, Merck, Darmstadt, Germany. A SWCNT aqueous dispersion (solid content, 0.2 wt.%) was purchased from Meijo Nano Carbon Co., Ltd., Aichi, Japan. A PEDOT:PSS aqueous dispersion, Clevios PH1000, was purchased from Heraeus Deutschland GmbH & Co., Leverkusen, Germany. Tetrahydrofuran (THF) with a stabilizer and 1,4‐butanediol (BG) (> 98.0%, Wako Special Grade) was purchased from Fujifilm Wako Pure Chemical Corp., Osaka, Japan. The Capstone FS‐31 fluorosurfactant was purchased from Chemours Co., Wilmington, DE, USA.

### 4.2 Fabrication of the SWCNT and PEDOT:PSS Nanofilms

All thin films were fabricated using a roll‐to‐roll gravure coating system (Mini‐labo, Yasui Seiki, Kanagawa, Japan). A PVA solution (5 or 7 wt.%) was coated on a PET film (Lumirrors L‐25T60, Toray Industries Inc., Tokyo, Japan) and dried at 80°C. A SBS solution (1, 2.5, or 5 wt.%) in THF was then coated on the PET/PVA film using the same method. The SWCNT dispersion containing 0.01 wt.% FS‐31 and the PEDOT:PSS dispersion containing 5 wt.% BG and 1 wt.% FS‐31 were stirred separately using a magnetic stirrer at room temperature for 24 h. Subsequently, the SWCNT and PEDOT:PSS dispersion were coated on separate PET/PVA/SBS films and dried at 80°C [34]. Finally, the PVA/SBS/SWCNT and PVA/SBS/PEDOT:PSS films were peeled off of the PET substrates using a tape frame method, followed by immersion in deionized water to dissolve the sacrificial PVA layer (Figure S1). The nanofilm was then transferred onto leaf surfaces using nylon mesh [22].

### 4.3 Characterization of the Structural Properties

The surface morphologies of the thin films attached to Si wafers were observed by field‐emission SEM (S‐5500, Hitachi High‐Tech Corporation, Tokyo, Japan) using an acceleration voltage of 10 kV [34]. The thicknesses of the thin films were measured using a stylus profilometer (Dektak XT, Bruker, Billerica, MA, USA). UV‒vis spectra of the fabricated thin films attached to a quartz substrate were measured in the wavelength range of 190–1100 nm using a GENESYS 50 spectrophotometer (Thermo Fisher Scientific, Waltham, MA, USA).

### 4.4 Characterization of the Electrochemical Properties

SWCNT nanofilm, PEDOT:PSS nanofilm, and commercial hydrogel electrodes (Vitrode F‐150M, Nihon Kohden, Tokyo, Japan) were attached to both sides of the leaf's central vein. Each electrode area was 15 mm × 20 mm with 1 cm between electrodes. Electrochemical impedance spectroscopy was performed using a potentiostat (SP‐300, BioLogic, Grenoble, France) in voltage mode (PEIS), sweeping from 0.1 Hz to 10 kHz. The amplitude of the applied voltage was set to 1000 mV.

### 4.5 Monitoring Plant Electrophysiology Signals

LIB changes in pothos (*Epipremnum aureum*) and soybean (*Glycine max*) leaves were measured using a digital multimeter (34461A, 34465A, Keysight Technologies, Santa Rosa, CA, USA) at a sampling rate of 1 Hz, and the white LEDs in the darkroom were turned on and off every 2 h (Figure 1e,f). The fabricated conductive nanofilm electrodes (70–480 nm, 15 mm × 20 mm area) were attached to both sides of the leaf vein with 1 cm between the electrodes. A flexible Cu‐coated polyimide film (Metaloyal (polyimide layer, 25 µm; Cu layer, 8 µm), Toray Industries Inc., Tokyo, Japan) and gold wire (φ 0.15 mm, Nilaco Corp., Tokyo, Japan) were used as the collector and interconnect materials. The reference electrode on the leaf was covered with Al foil to shield it from light irradiation. The LIB signal was measured at a photon density of 40–50 µmol/m^2^s and a temperature of 20–25°C. A photometer (SE‐MQ‐500, Apogee, Logan, UT, USA) was used to measure the photon density.

### 4.6 Characterization of the Conformability to Trichome‐Bearing Surfaces

The thin films were attached to trichome‐bearing soybean leaves to evaluate their adhesion to the uneven leaf surface. SWCNT nanofilms with thicknesses of 480, 320, and 70 nm were applied to soybean leaves; LIB changes were compared on Days 1 and 14. Images were captured using a stereomicroscope (MS‐200, Asahikogaku, Tokyo, Japan). The surface morphologies were observed at higher magnification by SEM (JCM‐6000, JEOL Ltd., Tokyo, Japan) using acceleration voltages of 5 and 10 kV. For SEM imaging, the sectioned samples were immersed in liquid nitrogen and transferred to a freeze‐drier (VFD‐03, AS ONE Corporation, Osaka, Japan). The samples were freeze‐dried for 24 h, and the surface of the leaf was coated with Au by sputtering for 30 s. The impedance of each electrode was measured using the same procedure described in “Characterization of the electrochemical properties.”

### 4.7 Characterization of the Long‐Term Durability

LIB changes were compared immediately after electrode application (Day 1) and 14 days later (Day 14) using SWCNT nanofilm electrodes and conventional electrodes. Opaque hydrogel electrodes (Vitrode F‐150M), transparent electrodes (Vitrode F‐150U3, Nihon Kohden, Tokyo, Japan), and disk electrodes (NE‐118A, Nihon Kohden, Tokyo, Japan) were used as conventional electrodes. A conductive paste (ZV‐181E, Nihon Kohden, Tokyo, Japan) was used to fix the disk electrodes. The physical condition of the electrode application site was also compared between Days 1 and 14 in pothos, soybean, and tobacco leaves. The SNR (in dB) of LIB was calculated using the following equation:

(2) $$\begin{array}{c} SNR=20\log_{10}\frac{A_{s}}{A_{n}} \end{array}$$

where *A*_s_ and *A*_n_ are the LIB amplitude (Δ*V*) between P1 and P2, and the standard deviation of the background noise, respectively. To accurately evaluate *A*_s_ without contamination from LIB signals duration, the background period for calculating *A*_s_ was defined as the 20 min immediately preceding the LED ON and LED OFF event.

### 4.8 Detection of Photosynthesis Inhibition

DCMU (Nacalai Tesque, Kumamoto, Japan) was used as a photosynthesis inhibitor in the pothos leaf. DMSO (Wako Pure Chemical Corp., Osaka, Japan) was used to dissolve DCMU. Pothos was placed in darkness for at least 30 min before DCMU treatment. Dark‐acclimated leaves were wrapped in paper towels soaked in DCMU solution (400 µm) for approximately 27 h. DCMU was dissolved in DMSO and then mixed with deionized water to keep the DMSO concentration less than 1% (v/v). The control solution consisted of DMSO at the same concentration, but without DCMU. Before LIB measurements, the treated leaf was dried in a dark room for 1 h to remove the effects of anaerobic conditions caused by the immersion treatment [41]. The LIB changes of the treated leaf were measured on Days 1 and 2 and again on Day 14. LIB amplitudes were calculated for each day and compared with the amplitude of the WT leaves, which was determined before DCMU treatment. Furthermore, the effect of DCMU treatment was confirmed by measuring chlorophyll fluorescence using a PAM MAXI imaging system (Walz, Effeltrich, Germany).

### 4.9 Statistics

The data were analyzed using Student's *t*‐test and one‐way analysis of variance with Tukey's test. Differences were assessed using a two‐sided test with an alpha level of 0.05.

## Author Contributions

Y.H., T.H., and T.F. conceived the ideas. Y.H. and T.H. fabricated and characterized the nanofilms. Y.H. performed biopotential measurements. Y.H. performed the detection of photosynthesis inhibition with the help of S.M. Y.H. wrote the manuscripts. T.F. Supervised this project. All authors discussed the results and commented on the manuscript.

## Funding

This work was supported by JSPS KAKENHI (grant numbers 21H03815, 24K21709, and 25H01215) from MEXT, Japan, Fusion Oriented REsearch for disruptive Science and Technology (FOREST) from Japan Science and Technology Agency (JST) (grant number JPMJFR203Q), the Samco Foundation, and Iketani Science and Technology Foundation. T. F. acknowledges the support from JST ASPIRE for Rising Scientists and Top Scientists (grant numbers JPMJAP2336 and JPMJAP2510).

## Conflicts of Interest

This work relates to the patent to be pended: JP2024‐131682. T.F. received honoraria from f‐Tech Co. Ltd. for another project.

## Supporting information




**Supporting File 1**: advs74781‐sup‐0001‐SuppMat.docx.

**Supporting File 2**: advs74781‐sup‐0002‐MovieS1.mp4.

**Supporting File 3**: advs74781‐sup‐0003‐MovieS2.mp4.

**Supporting File 4**: advs74781‐sup‐0004‐MovieS3.mp4.

## Data Availability

The data that support the findings of this study are available from the corresponding author upon reasonable request.
