# Supplementary material for: Pierceable, Water‐Resistant, and Transparent Nanofilm Electrodes Comprising Carbon Nanotubes for Long‐Term Monitoring of Plant Electrophysiology
Source: Adv Sci (Weinh). 2026 Mar 23;13(32):e22824. doi: 10.1002/advs.202522824 (PMC13252621; doi:10.1002/advs.202522824)
Supplement: Supplementary file 1 — Supporting File 1: advs74781‐sup‐0001‐SuppMat.docx. [file ADVS-13-e22824-s003.docx]

Supporting Information

**Pierceable, Water-Resistant, and Transparent Nanofilm Electrodes Comprising Carbon Nanotubes for Long-Term Monitoring of Plant Electrophysiology**

*Yusuke Hori, Tatsuhiro Horii, Shinji Masuda, Toshinori Fujie**

Y. Hori, T. Horii, S. Masuda, T. Fujie

Department of Life Science and Technology, Institute of Science Tokyo, Yokohama 226-8501, Japan

E-mail: t_fujie@life.isct.ac.jp

T. Fujie

Research Center for Autonomous Systems Materialogy (ASMat), Institute of Integrated Research (IIR), Institute of Science Tokyo, Yokohama 226-8501, Japan

T. Fujie

Institute of Biomedical Engineering (BME), Institute of Science Tokyo, Yokohama 226-8501, Japan


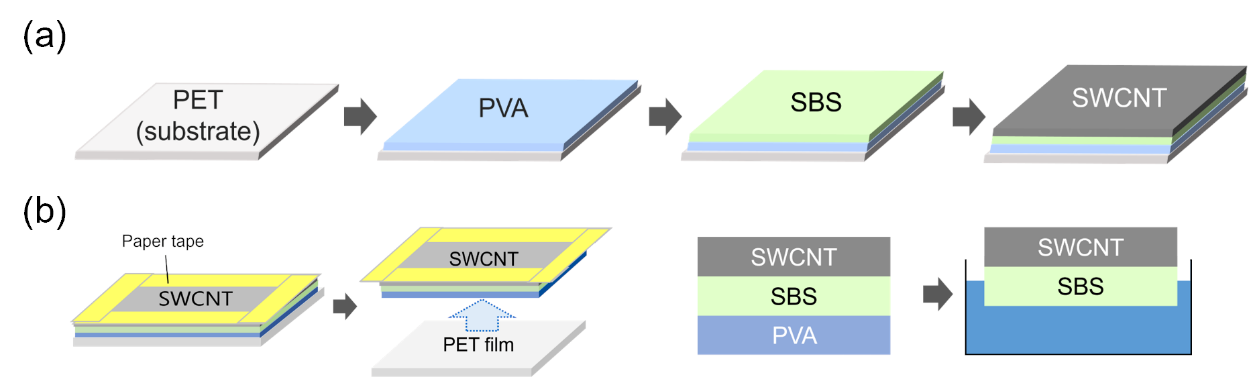


**Figure S1. Fabrication of the nanofilm electrode by gravure coating.** a, b) Illustration of the coating order used in the gravure coating method (a) and the tape frame method, showing the peeling and washing steps (b).


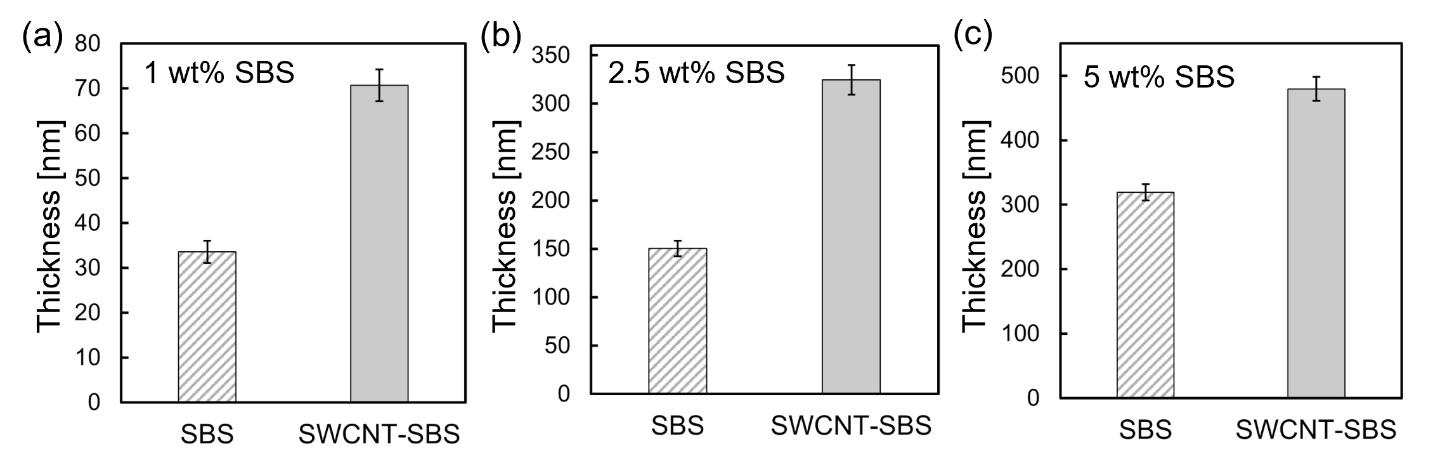


**Figure S2. Thicknesses of the SBS and SWCNT nanofilms.** a–c) Measured film thicknesses of SWCNT nanofilms with total thicknesses of 70 nm (a), 320 nm (b), and 480 nm (c), along with the corresponding SBS layer thicknesses. The thicknesses of the SWCNT layers, calculated by subtracting the SBS thickness from the total film thickness, are 40 nm (a), 170 nm (b), and 160 nm (c), respectively. Error bars denote the standard error.


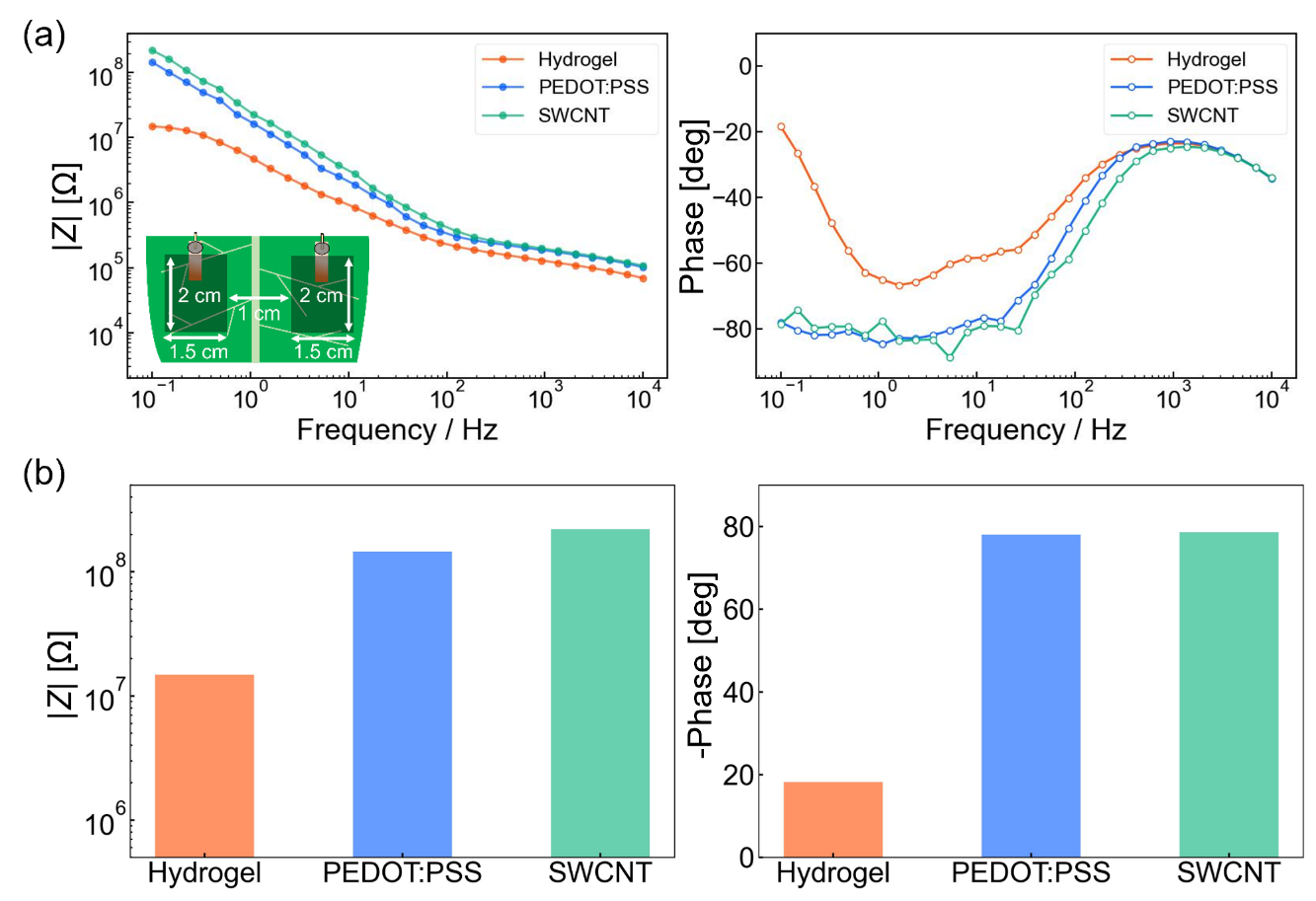


**Figure S3. EIS characterization of the hydrogel, PEDOT:PSS nanofilm, and SWCNT nanofilm electrodes.** a) Bode plots showing the impedance magnitude and phase response of hydrogel, PEDOT:PSS nanofilm, and SWCNT nanofilm electrodes attached to both sides of the leaf’s central vein, showing increasing impedance with decreasing frequency. Each electrode is 15 mm × 20 mm, with a 10 mm inter-electrode spacing. b) Bar graphs comparing the impedance and phase of the hydrogel, PEDOT:PSS nanofilm, and SWCNT nanofilm electrodes at 0.1 Hz. The |Z| value of the SWCNT nanofilm electrode was 2.21 × 10^8^ Ω, comparable to that of the PEDOT:PSS nanofilm electrode (1.45 × 10^8^ Ω). The hydrogel electrode showed an impedance of 1.49 × 10^7^ Ω, one order of magnitude smaller than the nanofilm electrodes, suggesting that the contact impedance of the conductive nanofilms is higher than that of the hydrogel electrode. The hydrogel electrode showed a phase angle of −18°, indicating predominantly resistive behavior at low frequencies. In contrast, the PEDOT:PSS nanofilm and SWCNT nanofilm electrodes exhibited phase angles of −78° and −79°, respectively. These findings indicate that capacitive effects dominate in the low-frequency range, which explains the higher contact impedance of both the SWCNT nanofilm and PEDOT:PSS nanofilm electrodes, compared with the conventional hydrogel electrodes.


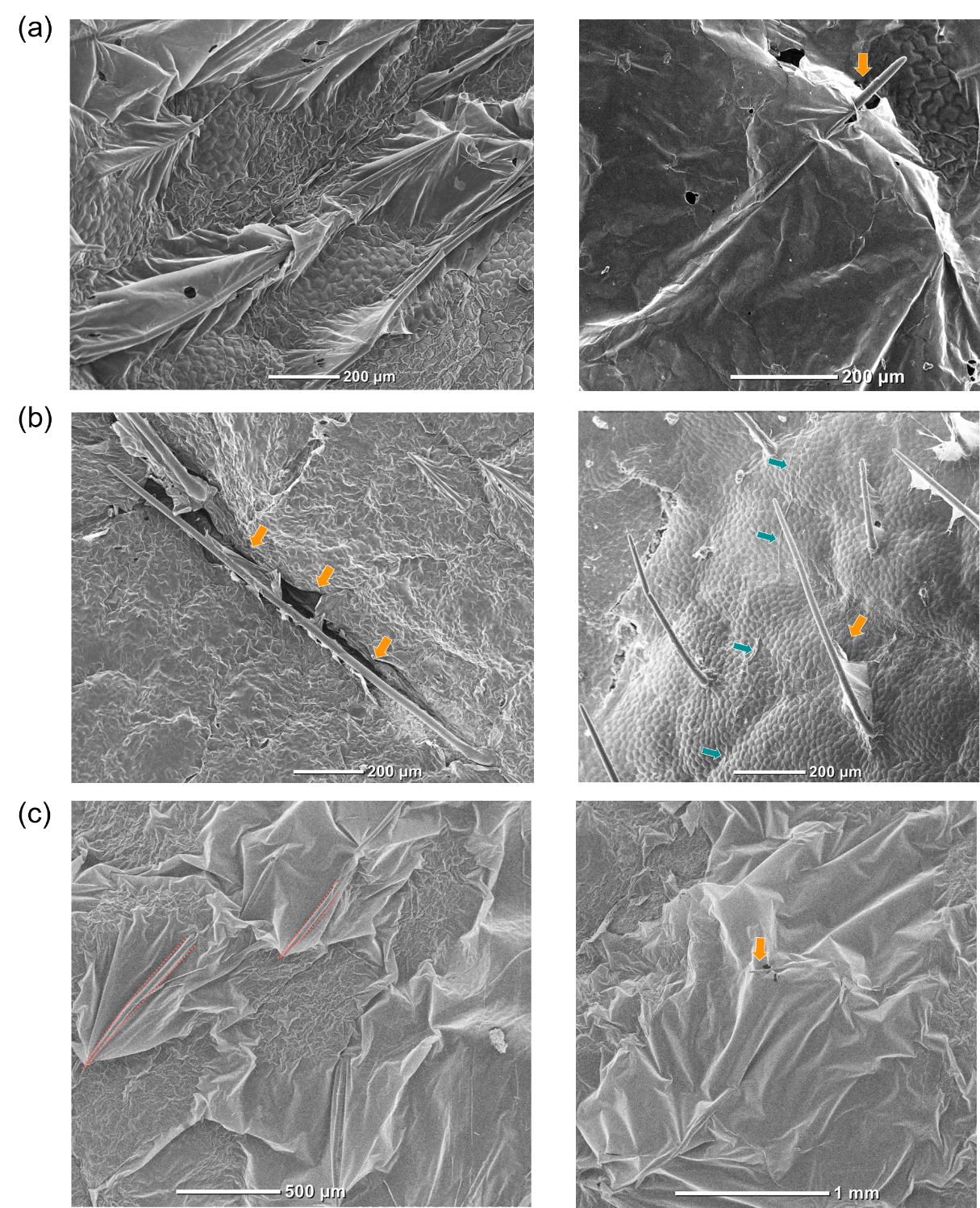


**Figure S4. SEM images of thin-film electrodes attached to soybean leaves.** 320 nm SWCNT nanofilms (a), 70 nm SWCNT nanofilms (b), and PEDOT:PSS nanofilms (c). Orange arrows highlight the places where the films are pierced by trichomes. a) The tips of some trichomes pierce the 320 nm SWCNT nanofilm, and portions of the film are attached to the leaf surface. b) The 70 nm SWCNT nanofilm conforms more extensively to the leaf surface owing to localized tearing caused by trichomes. The right panel is an enlarged view of Fig. 2f. Blue arrows indicate the border of the nanofilm (right area) and the leaf (left area), exhibiting the high conformability of the film to the leaf surface. c) The images indicate that the PEDOT:PSS nanofilm envelops the trichomes. Red dashed lines indicate trichomes. Several trichomes pierce the film, but some areas floated away from the leaf surfaces.


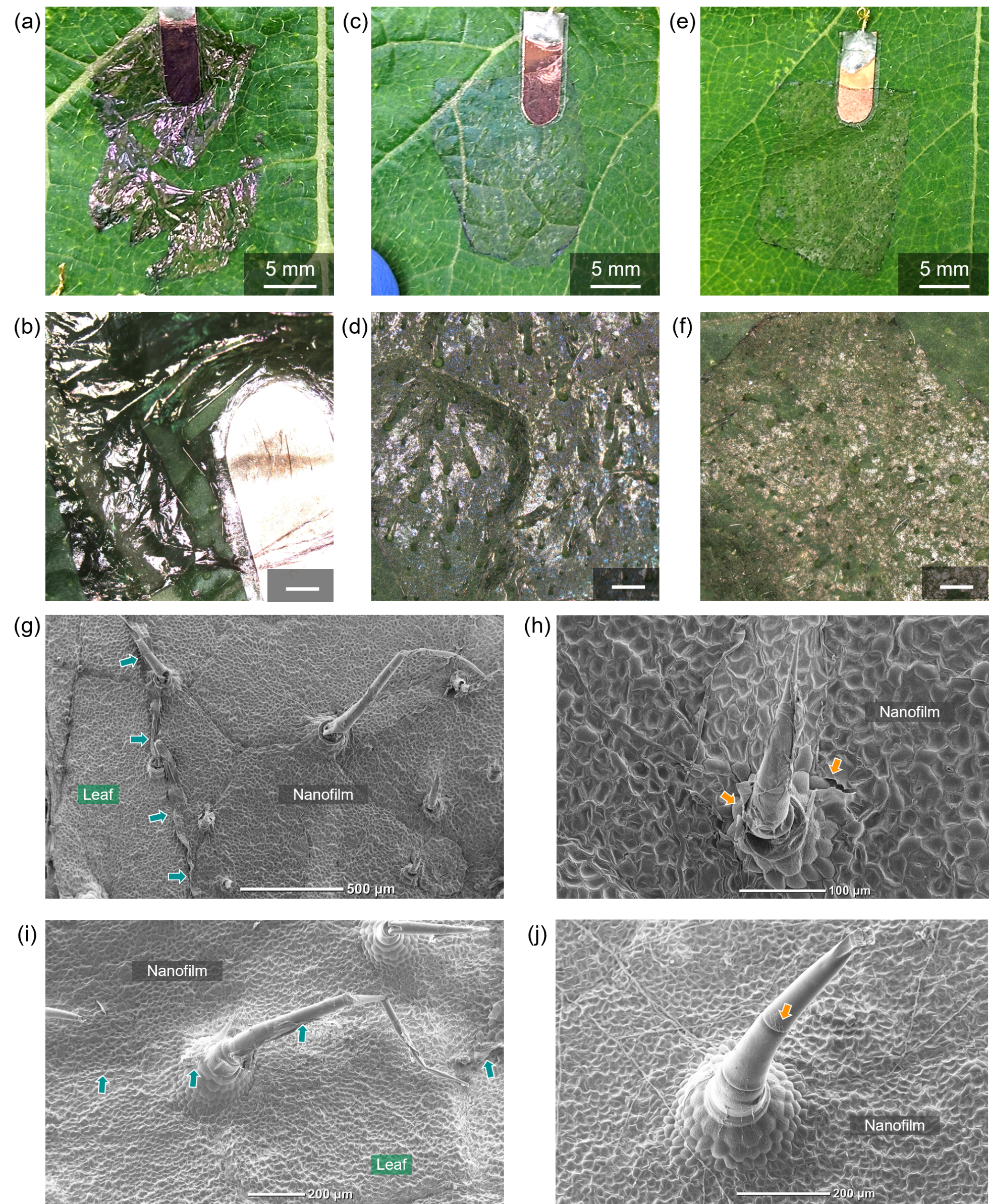


**Figure S5. Conformability of SWCNT nanofilms attached to pumpkin leaves.** a, b) Macroscopic Photograph (a) and microscopic image (b) of the 480 nm SWCNT nanofilm attached to a leaf. 480 nm films floated on the leaf surface due to trichomes, and the film was broken after drying. c, d) Macroscopic Photograph (c) and microscopic image (d) of the 320 nm SWCNT nanofilm attached to a leaf. e, f) Macroscopic Photograph (e) and microscopic image (f) of the 70 nm SWCNT nanofilm attached to a leaf. g, h) SEM images of the 320 nm SWCNT nanofilm attached to a pumpkin leaf. i, j) SEM images of the 70 nm SWCNT nanofilm attached to a pumpkin leaf. The film conforms extensively to the leaf surface. Blue arrows indicate the boundary between the leaf surface and the nanofilm. Orange arrows indicate locations where the trichomes partially pierced the nanofilm. The scale bars in the microscopic images (b, d, f) represent 1 mm.


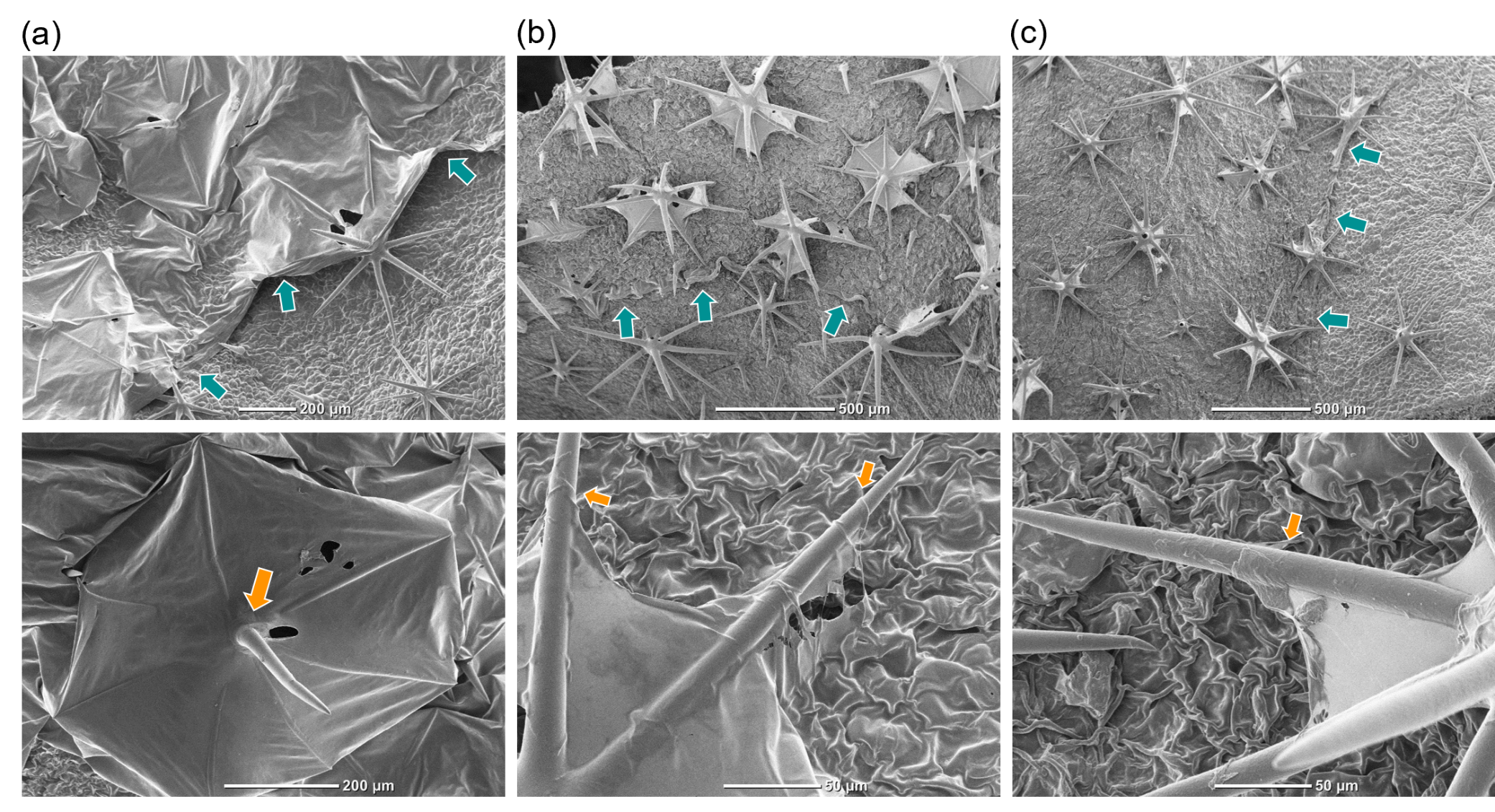


**Figure S6. Conformability of SWCNT nanofilms attached to eggplant leaves.** a) SEM images of the attached 400 nm SWCNT nanofilm. The film showed significant lifting due to trichomes, resulting in a large area of the nanofilm failing to achieve sufficient contact with the leaf surface. b) SEM images of the attached 320 nm SWCNT nanofilm. Even the horizontally oriented trichomes characteristic of eggplant leaves pierced the film. However, the film partially detached around the piercing sites, forming an umbrella-like shape. c) SEM images of the attached 70 nm SWCNT nanofilm. The 70 nm films achieved conformal adhesion over a much wider area with minimized umbrella-shaped lifting. In each panel, the upper image shows an overview, and the lower image shows a magnified view of the trichome-piercing area. Blue arrows indicate the boundary between the leaf surface and the nanofilm, and orange arrows indicate locations where the trichomes partially pierced the nanofilm.


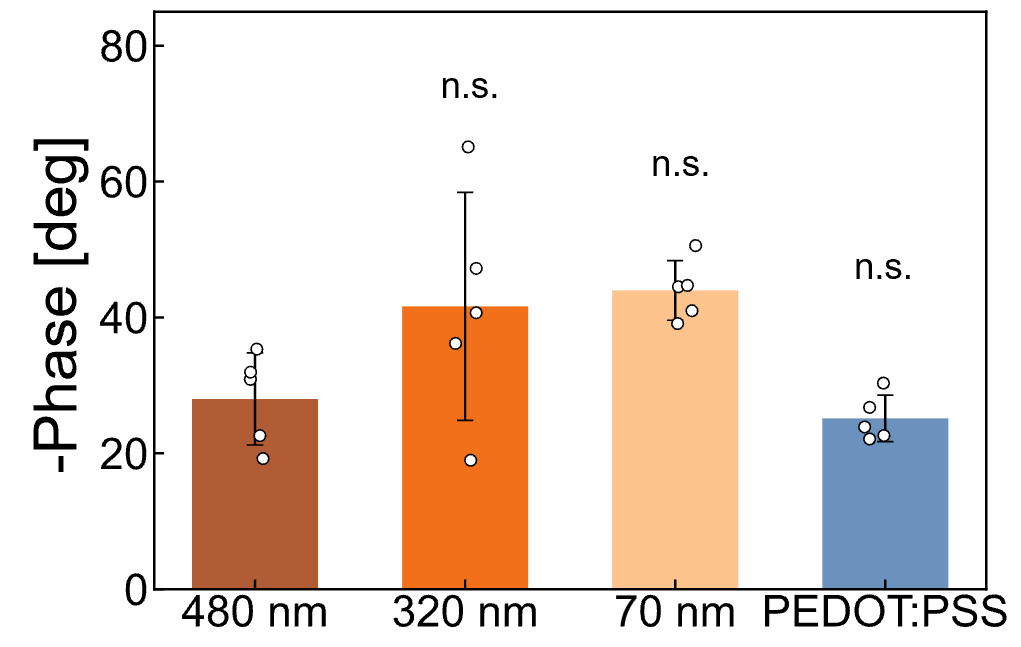


**Figure S7. Bar graphs comparing the phase.** Values are the means ± standard deviation of five independent experiments. One-way analysis of variance with Tukey’s test for the 480 nm SWCNT nanofilm versus the 70 nm SWCNT nanofilm, 320 nm SWCNT nanofilm, and PEDOT:PSS nanofilm; ns, no significant difference. The 70 nm SWCNT nanofilms showed a slightly lower phase angle than the 480 nm films, indicating that smaller air gaps lead to higher capacitance of the electrode–tissue interface.


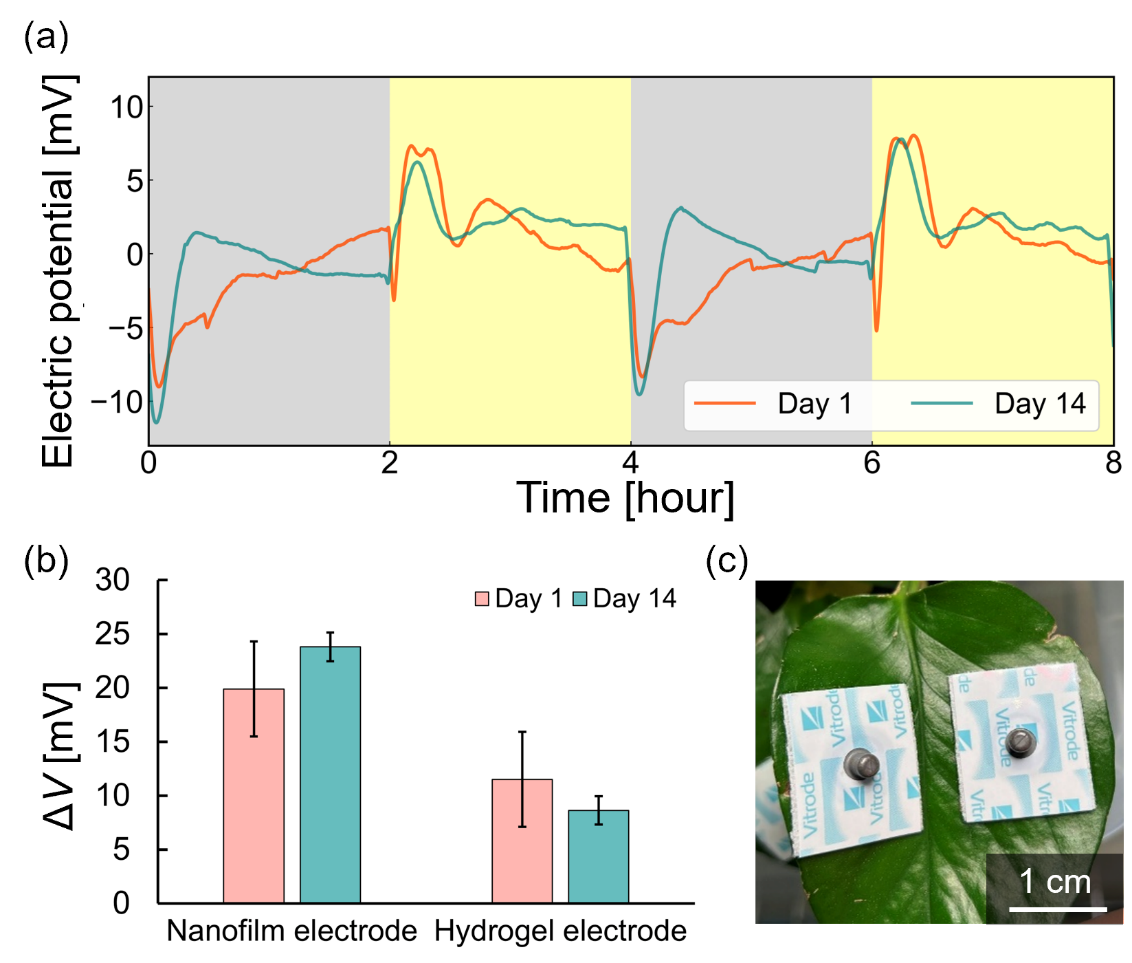


**Figure S8. Non-invasiveness of hydrogel electrodes in long-term measurements.** a) LIB signals for hydrogel electrodes during two cycles of illumination and dark periods on Days 1 and 14. b) Comparison of LIB amplitudes for hydrogel and SWCNT nanofilm electrodes on Days 1 and 14. Error bars denote the standard deviation. Minor differences in LIB waveforms were observed between Days 1 and 14. Specifically, the LIB acquired by hydrogel electrodes displayed a bifurcated depolarization phase (two distinct P2s) on Day 14. These variations may be attributed to differences in the plant’s moisture content and fluctuations in ambient temperature and humidity during the experiments. In addition, the LIB amplitude measured using the SWCNT nanofilm electrodes was approximately twice that of the hydrogel electrodes. This increase is due to the higher transmittance of the SWCNT nanofilm electrodes (Fig. 1d), which increases the intensity of the light reaching the leaf surface and enhances the biopotential response.^[1]^ c) Images of a pothos leaf after attachment of the hydrogel electrode for 2 weeks. The hydrogel electrode exhibited non-invasiveness.


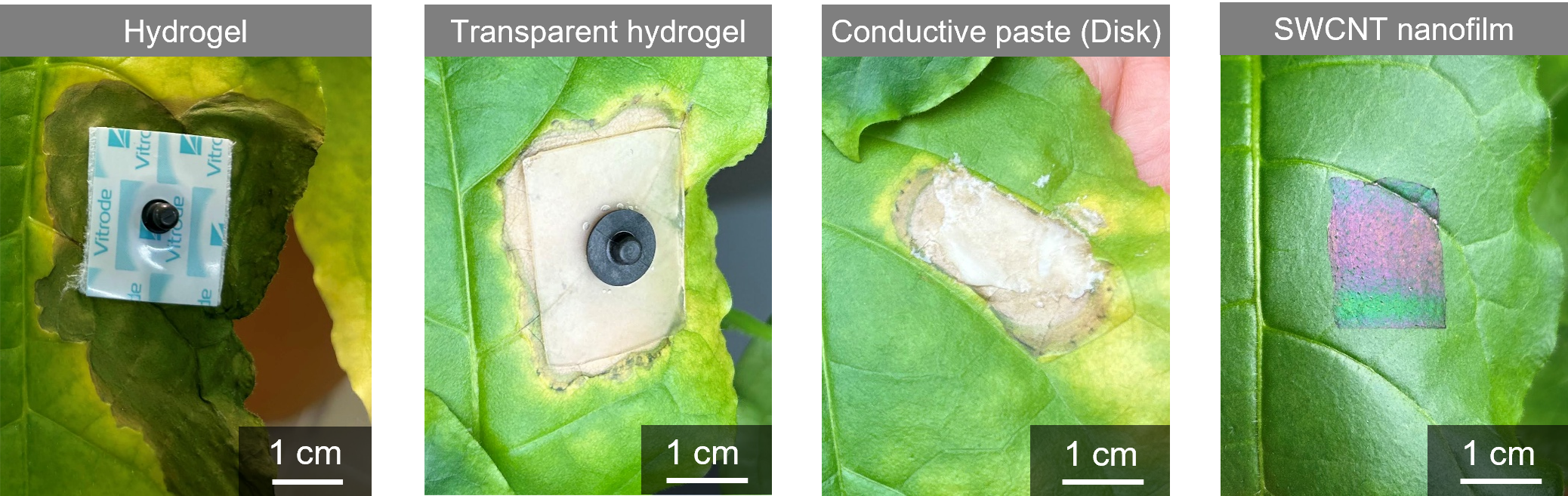


**Figure S9. Pictures of tobacco leaves after attachment of the opaque hydrogel electrode, transparent hydrogel electrode, conductive paste for disk electrode, and SWCNT nanofilm electrode for 2 weeks.** The attachment area of the hydrogel and disk electrodes showed invasiveness. The SWCNT nanofilm electrode showed non-invasiveness.


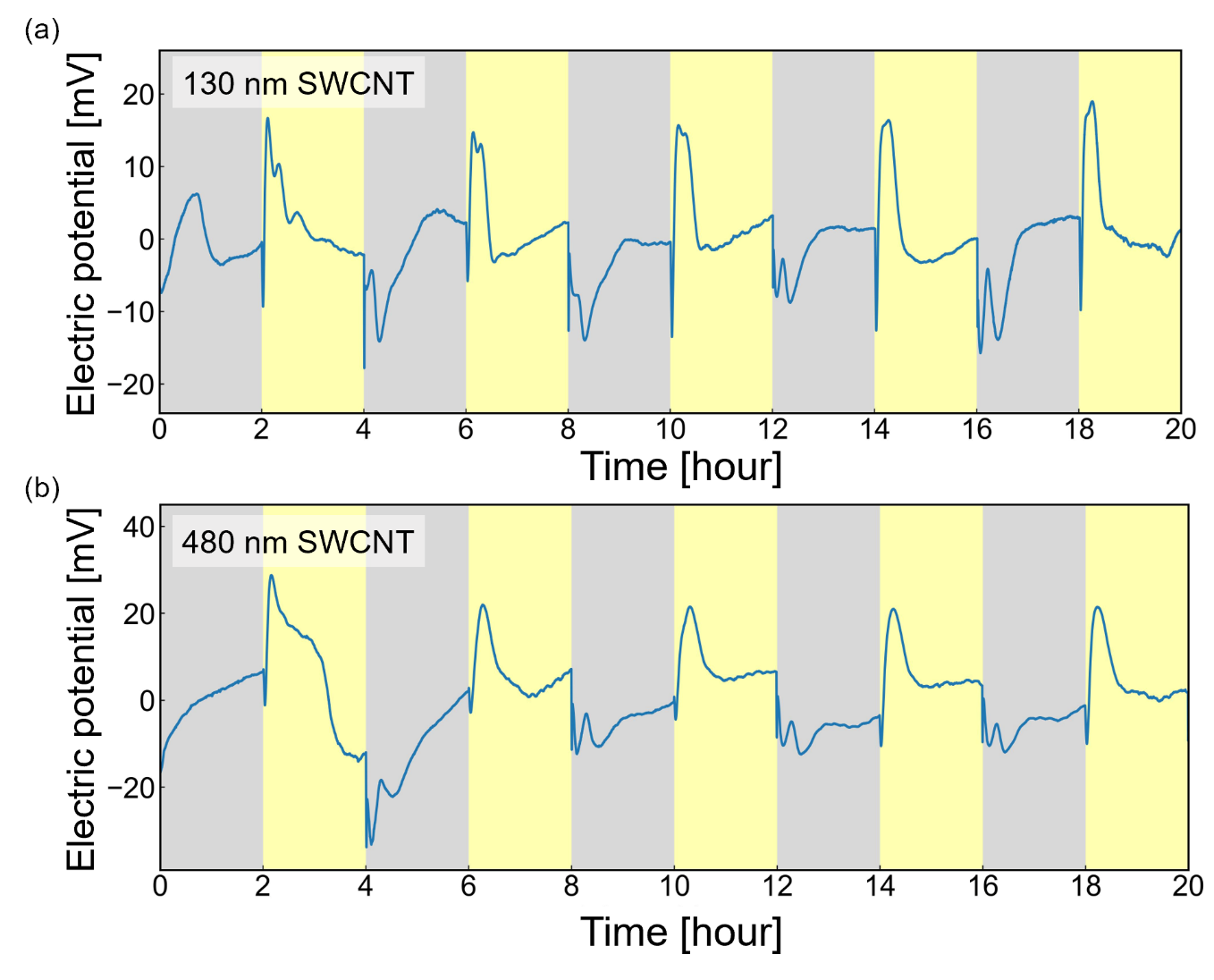


**Figure S10. LIB signals recorded 1 month after the attachment of SWCNT nanofilm electrodes.** a) LIB measured using 130 nm SWCNT nanofilm electrodes. b) LIB measured using 480 nm SWCNT nanofilm electrodes.


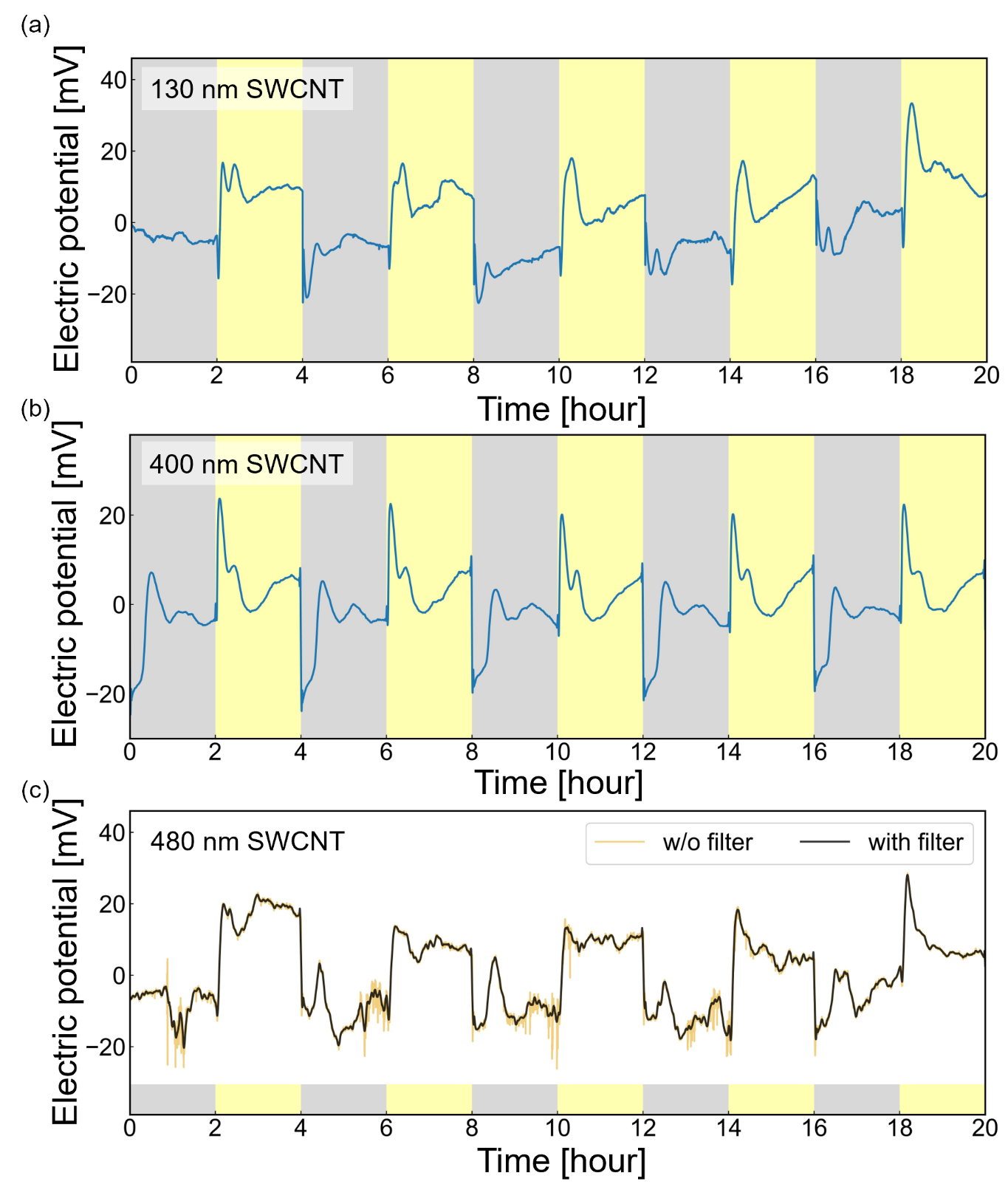


**Figure S11. LIB signals recorded 2 months after the attachment of SWCNT nanofilm electrodes.** a, b) LIB measured by 130 nm SWCNT nanofilm electrodes (a) and 400 nm SWCNT nanofilm electrodes (b). c) Comparison of the raw LIB waveform (without signal processing) and the processed waveform (smoothed with a Savitzky-Golay filter) recorded using 480 nm SWCNT nanofilm electrodes.


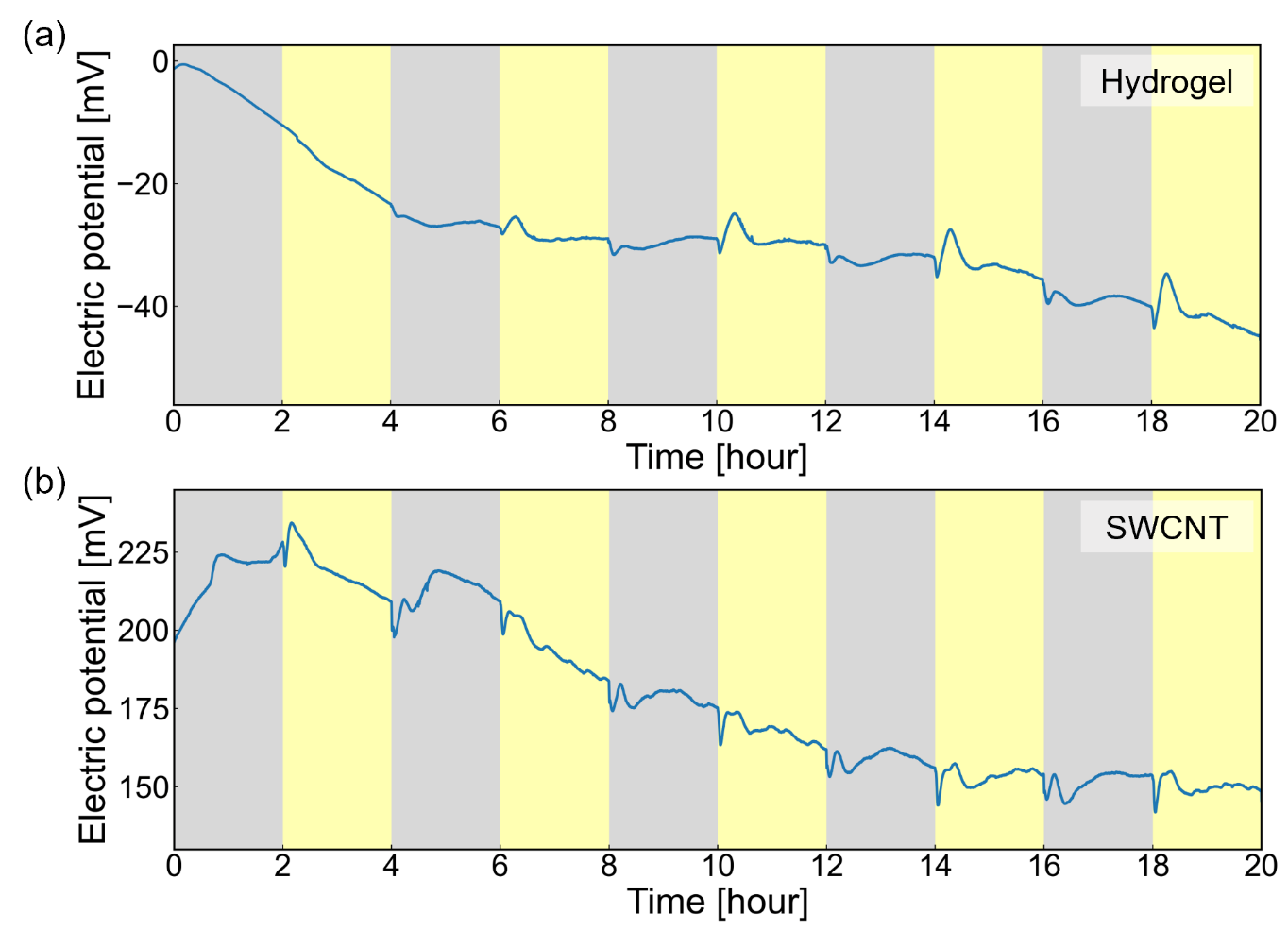


**Figure S12. LIB measurements after the water flow test.** LIB measured using hydrogel electrodes (a) and SWCNT nanofilm electrodes (b) without signal processing.


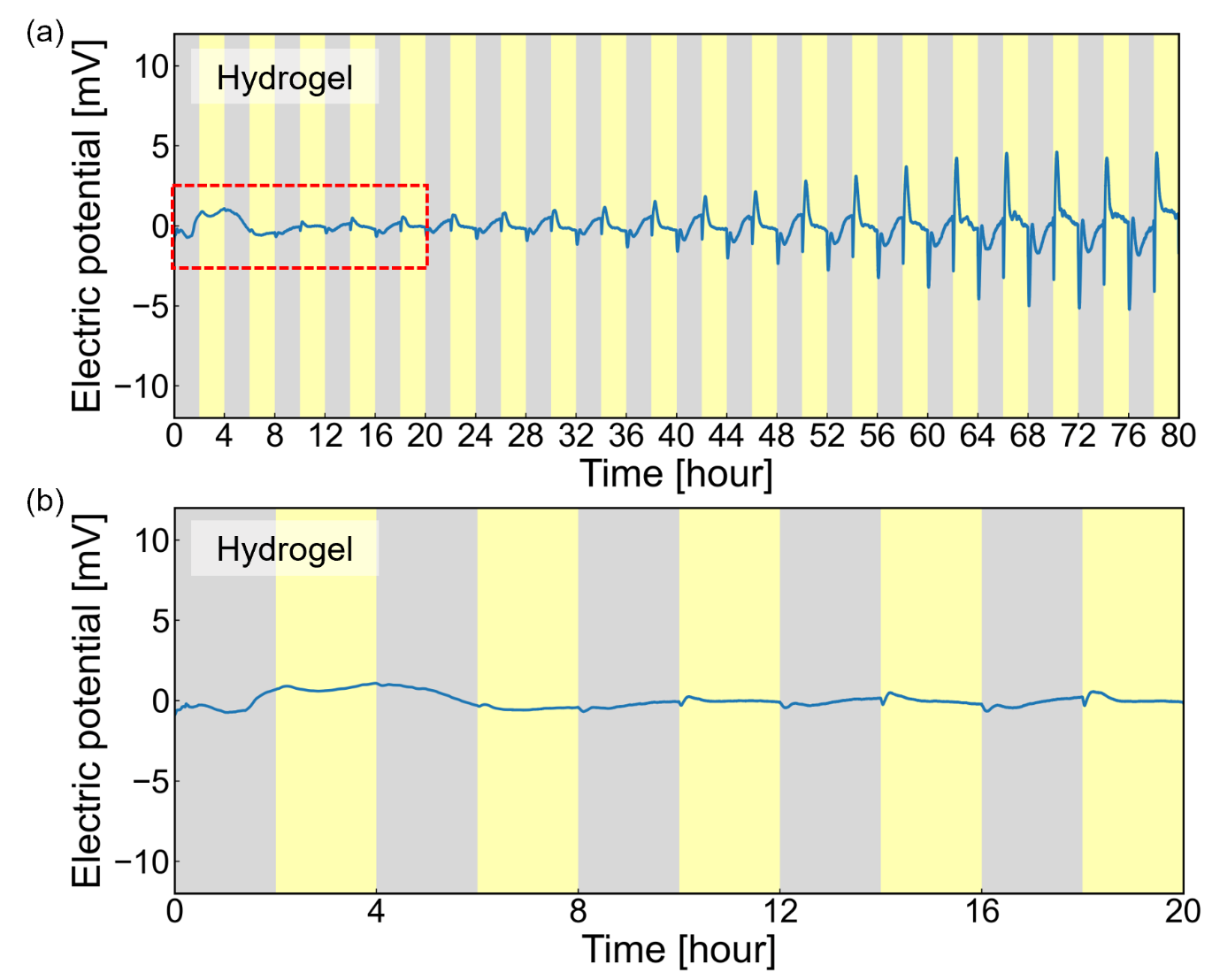


**Figure S13. LIB measurements using hydrogel electrodes following the 30-minute immersion test.** a) Continuous LIB monitoring for 80 hours immediately following the immersion test. The data indicate a complete failure of LIB measurement right after immersion (particularly within the first 2 hours). However, the LIB amplitude steadily recovered as the swollen hydrogel gradually dried. b) Enlarged view of the extracted LIB data from Figure S13a, focusing on the period from 0 to 20 hours after the immersion test.


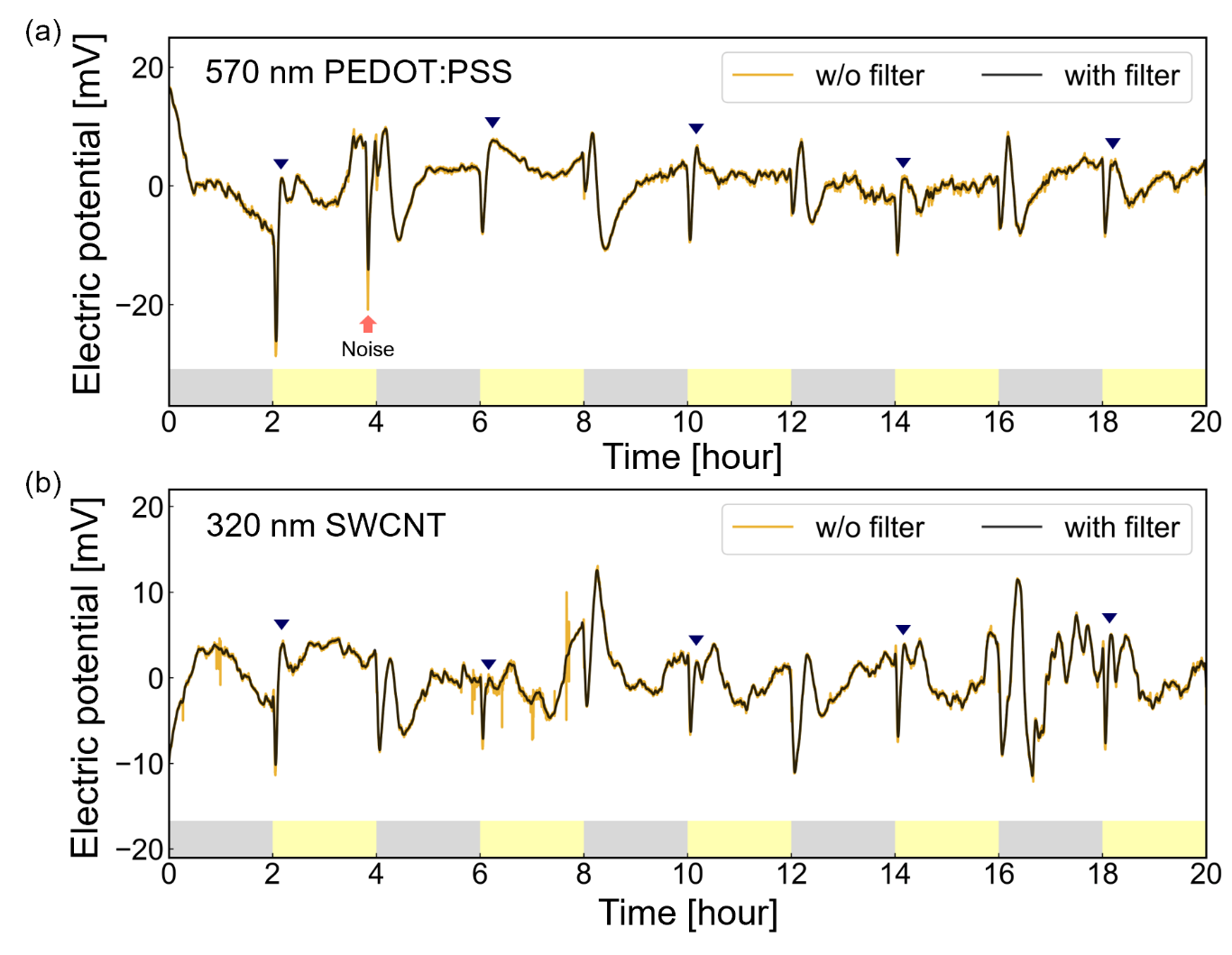


**Figure S14. LIB measurements using PEDOT:PSS and SWCNT nanofilm electrodes following the 30-minute immersion test.** a, b) Continuous LIB monitoring for the first 20 hours using PEDOT:PSS nanofilms (a) and SWCNT nanofilms (b). Although both electrodes exhibited slight noise, distinct LIB peaks were clearly observed even immediately after the immersion test. The acquired LIB signals are denoted by blue triangles.


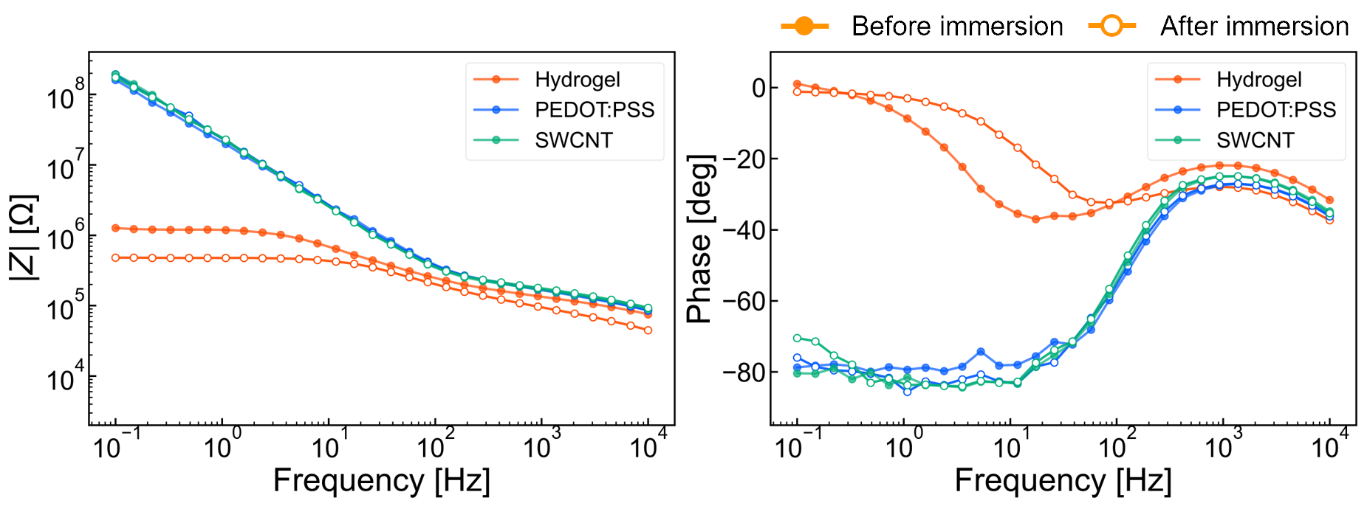


**Figure S15. Bode plot showing the impedance magnitudes and phase responses before and after the 30-minute immersion test using hydrogel electrodes, PEDOT:PSS nanofilms, and SWCNT nanofilms.**


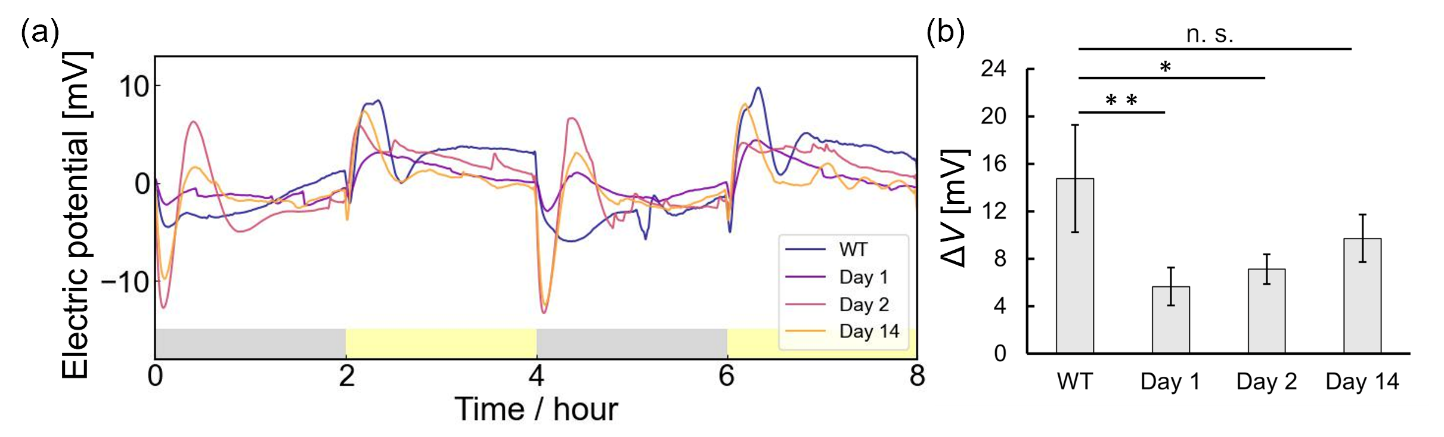


**Figure S16. Photosynthesis inhibition detected using hydrogel electrodes.** a) LIB measurements of WT, Day 1, Day 2, and Day 14 using hydrogel electrodes. b) LIB amplitudes for each day. The Student’s t-test was used to compare two groups. Error bars denote standard deviation. **p* < 0.05, ***p* < 0.01; ns, no significant difference.


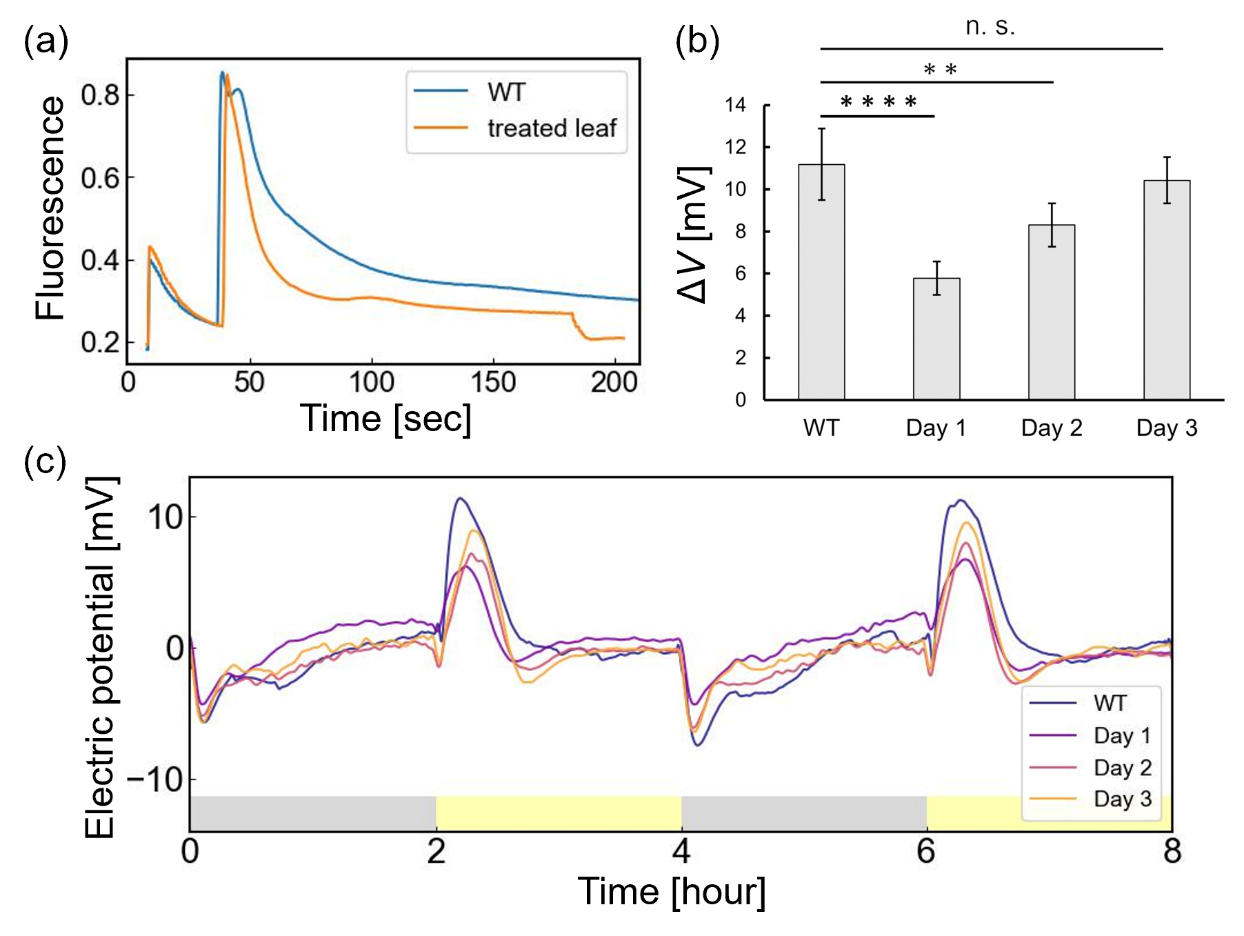


**Figure S17. Control test for the detection of photosynthesis inhibition using hydrogel electrodes.** a) Relative fluorescence intensity of a leaf before treatment (WT) and immediately after treatment (treated leaf). Chlorophyll fluorescence was measured under an actinic light of 80 µmol/m^2^s. b, c) LIB measurements of WT, Day 1, Day 2, and Day 3 using hydrogel electrodes: amplitudes (b) and waveforms (c). The Student’s t-test was used to compare two groups. Error bars denote standard deviation. ***p* < 0.01, *****p* < 0.0001; ns, no significant difference.

**Supporting Movie**

**Movie S1:** Stability test of a conventional hydrogel electrode on a leaf surface under strong running water (simulated torrential rain) for 30 s. The hydrogel absorbs water and swells significantly.

**Movie S2:** Stability test of the SWCNT nanofilm electrode on a leaf surface under strong running water for 30 s. The nanofilm maintained robust adhesion to the leaf surface without delamination.

**Movie S3:** Stability test of a PEDOT:PSS nanofilm electrode on a leaf surface under strong running water for 30 s. The strong water flow easily washed away the electrode, demonstrating its limited durability against heavy rainfall.

**Table S1. Comparison of recent plant surface electrodes**

| **Sensing material** | **Substrate** | **Thickness** | **Conformability to trichomes** | **Transmittance** | **Gas permeability** | **Water resistance** | **Long-term stability** | **Ref.** |
| --- | --- | --- | --- | --- | --- | --- | --- | --- |
| SWCNT | SBS | 70–480 nm | Yes (trichome-piercing) | 80% | 28,316 g m^-2^ (2h)^-1^ (WVTR) | Yes  (water flow / immersion) | > 2 months measurement  (up to 10 months measurement) | This work |
| poly(PEG/PPG/PCL/urethane) thermogel (NaCl) | N/A | 51.9 μm  (dehydrated gel) | Yes (morphologically of hydrogel) | Not reported (Low transmittance due to thick CNT-BC film on the gel) | 488 g m^-2^ (day)^-1^  (water vapor permeability of dehydrated gel) | Not reported  (Hydrogel-based electrodes lack water resistance) | ~1 month measurement (dehydrated gel) | [2,3] |
| AgNW | N/A | 100 nm | Yes  (mechanism is unclear) | 80% | Yes (not blocking stomatal pores) | Not reported | ~14 days measurement  (~60 days attachment) | [4] |
| PEDOT:PSS | N/A | ~500 nm | Not reported | Not reported  (~80% typical for PEDOT:PSS) | Not reported | Not reported | ~10 days measurement | [5] |
| PEDOT:PSS | Au/Parylene C | > 2 μm | Not reported | Not reported  (~80% typical for PEDOT:PSS) | Not reported | Not reported | Not reported | [6] |
| AgNW | PPG-mUPy+PU | 6–70 μm | Not reported | 60–70% | 275.5 L·S^−1^·m^−2^ (Air permeability) | Yes  (water flow) | ~15 days attachment | [7] |

**References**

[1] J. Li, Y. Yue, Z. Wang, Q. Zhou, L. Fan, Z. Chai, C. Song, H. Dong, S. Yan, X. Gao, Q. Xu, J. Yao, Z. Wang, X. Wang, P. Hou, L. Huang, Front. Plant Sci. 2019, 10.

[2] Yifei Luo, W. Li, Q. Lin, F. Zhang, K. He, D. Yang, X. J. Loh, X. Chen, Adv. Mater. 2021, 33, 2007848.

[3] Yi Jing Wong, Y. Luo, W. Li, E. V. L. Grate, F. Zhang, Z. Lv, Q. Lin, M. Zhang, Y. Miao, X. J. Loh, X. Chen, Science Advances 2026, 12, eady1400.

[4] T. He, J. Wang, D. Hu, Y. Yang, E. Chae, C. Lee, Nat. Commun. 2025, 16, 3244.

[5] F. Meder, S. Saar, S. Taccola, C. Filippeschi, V. Mattoli, B. Mazzolai, Adv. Mater. Technol. 2021, 6.

[6] A. Armada-Moreira, A. M. Dar, Z. Zhao, C. Cea, J. Gelinas, M. Berggren, A. Costa, D. Khodagholy, E. Stavrinidou, Science Advances 2023, 9, eadh4443.

[7] M. Zhu, W. Xu, L. Chen, D. Wu, Z. Wang, X. Hu, X. Luo, R. Xiong, C. Huang, ACS Nano 2024, 18, 28834.
